# Multimodal image registration and connectivity analysis for integration of connectomic data from microscopy to MRI

**DOI:** 10.1038/s41467-019-13374-0

**Published:** 2019-12-03

**Authors:** Maged Goubran, Christoph Leuze, Brian Hsueh, Markus Aswendt, Li Ye, Qiyuan Tian, Michelle Y. Cheng, Ailey Crow, Gary K. Steinberg, Jennifer A. McNab, Karl Deisseroth, Michael Zeineh

**Affiliations:** 1 Department of Radiology, Stanford University, Stanford, CA 94035 USA; 2 Department of Bioengineering, Stanford University, Stanford, CA 94035 USA; 3 CNC Program, Stanford University, Stanford, CA 94035 USA; 4 Department of Neurosurgery and Stanford Stroke Center, Stanford University, Stanford, CA 94035 USA; 5 Department of Electrical Engineering, Stanford University, Stanford, CA 94035 USA; 6 Department of Psychiatry, Stanford University, Stanford, CA 94035 USA; 7 Howard Hughes Medical Institute, Stanford University, Stanford, CA 94035 USA

**Keywords:** Diffusion tensor imaging, Computational platforms and environments, Neural circuits, Neurological disorders

## Abstract

3D histology, slice-based connectivity atlases, and diffusion MRI are common techniques to map brain wiring. While there are many modality-specific tools to process these data, there is a lack of integration across modalities. We develop an automated resource that combines histologically cleared volumes with connectivity atlases and MRI, enabling the analysis of histological features across multiple fiber tracts and networks, and their correlation with *in-vivo* biomarkers. We apply our pipeline in a murine stroke model, demonstrating not only strong correspondence between MRI abnormalities and CLARITY-tissue staining, but also uncovering acute cellular effects in areas connected to the ischemic core. We provide improved maps of connectivity by quantifying projection terminals from CLARITY viral injections, and integrate diffusion MRI with CLARITY viral tracing to compare connectivity maps across scales. Finally, we demonstrate tract-level histological changes of stroke through this multimodal integration. This resource can propel investigations of network alterations underlying neurological disorders.

## Abstract (web summary)

Many approaches exist to process data from individual imaging modalities, but integrating them is challenging. The authors develop an automated resource that enables co-registered network- and tract-level analysis of macroscopic in-vivo imaging and microscopic imaging of cleared tissue.

## Introduction

Mapping neural networks is essential for understanding the functional signals underlying behavior and cognition^1^. For example, ischemic stroke is currently viewed, in part, as a disease of brain connectivity, as the region directly impacted by the infarct precipitates network-wide deficits in connected brain regions^2^. Previous studies have reported that, following cortical stroke, secondary remote degeneration can occur in connected regions such as the thalamus in the subsequent days and weeks^3–6^, which negatively affects functional outcomes^7–9^. Understanding how cellular changes evolve in connected regions is critical for understanding the progression of pathology and therefore for developing targeted interventions for stroke.

Examining the connections of these networks across the scales of measurement should enable the delineation of changes specific to disease states. Large-scale efforts are mapping rodent, primate, and human neural networks at both microscopic^10,11^ and macroscopic levels^12,13^, employing three-dimensional (3D) histology (e.g., clearing techniques), serial two-dimensional (2D) histology (e.g., connectivity atlases), and noninvasive in vivo imaging (e.g., diffusion magnetic resonance imaging (dMRI)). Recent advances in clearing techniques, such as CLARITY^14–17^, 3DISCO/iDISCO^18,19^, CUBIC^20^, and others^21,22^, enable the mapping of tracers, cell distributions, and processes in intact tissue. Tracing experiments with high-throughput serial 2D sections have been used to create connectivity atlases^10,11^. At the macroscopic scale, dMRI can noninvasively image larger fiber pathways across the brain and map changes associated with disease^23^. These approaches generate large datasets that require analysis tools to distill information about neural networks. While multiple approaches exist to process data from individual modalities, all of these require modality-specific expertise, and integrating across modalities presents a compound challenge.

First, existing pipelines for clearing techniques (Supplementary Table 1) employ segmentation of cellular features and registration to atlases to perform regional and whole-brain analyses such as cell counting^24–28^ but do not integrate with connectivity atlases and thus cannot readily assess changes across neural networks. In the context of disorders that involve disruption of neural networks, such as stroke, this lack of integration limits hypothesis-free assessments of upstream and downstream effects on connected regions across the brain. Similarly, quantifying histological features along individual fiber tracts within any particular network remains largely intractable, prohibiting the assessment of tract-level effects in disease.

Second, existing pipelines cannot systematically synthesize findings between 3D histology features and noninvasive in vivo imaging (e.g., MRI). Such a synthesis could identify and validate noninvasive imaging-based biomarkers to investigate within-animal longitudinal effects, which is not feasible using temporally sparse histological data. For example, direct comparison of histological features could reveal whether diffusion and relaxometry signal changes in MRI are due to demyelination, inflammation, edema, or other cellular processes. Moreover, such multimodal comparisons allow for the validation of lower-resolution imaging modalities. Putative tracts identified through dMRI tractography could be validated using virally labeled axonal fibers (observed with sub-micron resolution).

**Fig. 1:**
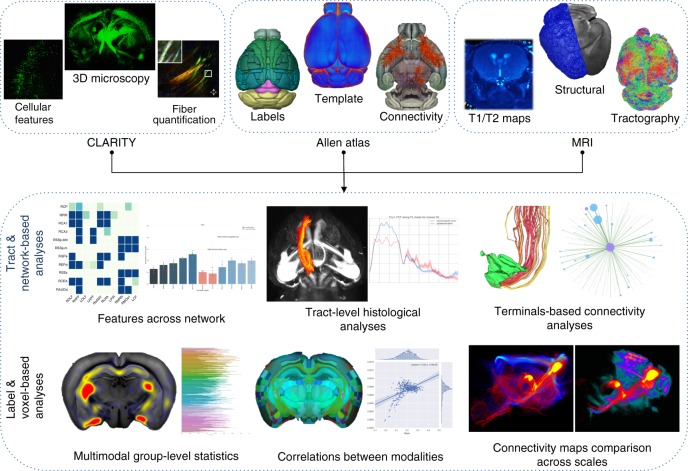
MIRACL enables the interrogation of brain pathways and cellular features across modalities. The resource integrates CLARITY data in the microscopic domain (top left) with macroscopic in vivo and ex vivo imaging data, such as structural MR, diffusion MR, and quantitative MR relaxometry (top right), in the Allen atlas reference frame “ARA” (top center). This integration enables tract and network-based analyses such as studies of histological features across network graphs or along fiber tracts and connectivity analyses based on projection terminals. This pipeline also performs group-level statistics, multimodal correlations, as well as comparisons of connectivity maps across scales.

To address these opportunities, we present the Multimodal Image Registration And Connectivity anaLysis (MIRACL) pipeline. This automated, open-access resource enables the co-registered analysis of both macroscopic in vivo imaging as well as microscopic imaging of cleared tissue. Through direct integration with connectivity atlases in the standard Allen atlas reference frame, MIRACL can investigate pathological features spanning multiple networks based on study-specific lesion maps. Our resource further allows analyses of histological and imaging features along fiber pathways to discern tract-level alterations (Fig. 1, Supplementary Table 1). Integration of computed tomography (CT), structural, dMRI^29^, and quantitative MRI^30,31^ supports the translation to in vivo measurements and localization of effects to specific brain regions. By combining in vivo and ex vivo dMRI, CLARITY viral tracing, and the Allen connectivity atlas, we can further inform and validate in vivo diffusion tractography.

We test MIRACL in multiple applications in the context of assessing global structural and network changes following ischemic stroke. First, we analyze group-wise cellular changes at the acute stage of experimentally induced stroke and correlate them with in vivo imaging. Second, we assess the effects of stroke on areas structurally connected to the ischemic core. Third, to enhance the accuracy of connectivity mapping (i.e., defining the strength of connections), we develop a method to compute connectivity based on terminating projections from CLARITY viral injections. Fourth, to compare connectivity maps at different scales, we investigate the medial prefrontal cortex (mPFC) projections by contrasting dMRI, CLARITY-optimized viral tracing, and a connectivity atlas. Finally, we use ultra-high-resolution dMRI tractography and projections from CLARITY viral tracing to identify tract-level changes resulting from stroke.

## Results

### Experimental procedures, design, and validation

The presented suite combines developed and existing analysis techniques and algorithms to integrate these multiple modalities and enable the investigation of pathological and tract-level effects on connected brain regions spanning multiple networks. For the mouse brain, we employed the widely used Allen Regional Atlas (ARA), including comprehensive ARA histologic images (Nissl staining), fine structure labels, and connectivity atlas^11^, though the pipeline can also accommodate other atlases for different species. We illustrate our pipeline’s capabilities using a model of ischemic stroke, a disorder in which a focal lesion can cause network-wide effects. We used 20 male Thy1-yellow fluorescence protein (YFP) mice, which brightly labels layer-specific motor and sensory neurons and axons^32^. Ten mice underwent transient middle cerebral artery occlusion (MCAO) and 10 littermates served as controls. All the stroke mice and 3 of the control group underwent in vivo MRI scanning (24 h after stroke) with high-resolution structural imaging, T1 and T2 mapping (quantitative maps of MRI relaxation times), and diffusion tensor imaging (DTI). The mouse brains were then extracted, immersed in an iodinated solution to enhance tissue contrast, and imaged ex vivo in a micro-CT. Following tissue clearing, the whole cleared mouse brains were scanned using a light-sheet microscope. To perform more detailed scanning of the stroke core (the area of infarcted tissue with acute ischemia), the brains were cut into coronal slabs (2–3 mm thick), and the slab with the core was further stained for propidium iodide (PI, a small nuclear marker) and scanned at a higher resolution (2.49 μm in-plane) on a confocal microscope. PI was chosen to complement the relatively sparse Thy1-YFP labeling for defining the stroke core since it is a more sensitive nuclear label (Supplementary Movie 1).

**Fig. 2:**
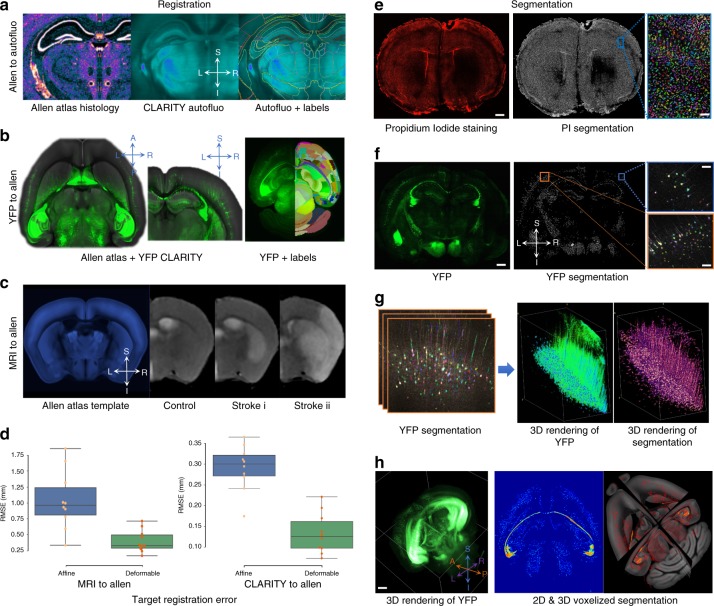
Validation highlighting MIRACL’s registration and segmentation accuracy. **a** High fidelity of CLARITY registration to the Allen Regional Atlas (ARA). Left: Coronal view of an ARA Nissl histology slice. Center: CLARITY auto-fluorescence channel of a representative stroke mouse registered to the ARA. Right: The same auto-fluorescence channel with overlaid label outlines (R–L: right–left, S-I: superior–inferior). **b** Axial and coronal views of a Thy1 yellow fluorescence protein (YFP) imaging volume (green) registered to the ARA template (grayscale) and an axial view of another registered CLARITY dataset with (right) and without (left) ARA labels. **c** Coronal views of three Allen-registered in vivo MR images (a control mouse, a striatal stroke mouse, and a cortico-striatal stroke, respectively). All mice in this study were scanned 24 h after stroke. **d** Low root mean squared error (RMSE) between transformed manually placed landmarks on the native MRI and CLARITY imaging volumes and ARA manually placed landmarks. Center line of box plot represents the median, bounds represent the first and third quantiles, and whiskers represent the lowest and highest datum within 1.5× the interquartile range of the lower and upper quantiles. **e** Segmentation results for nuclei using propidium iodide (PI) stain. Coronal view of a PI stroke brain and its corresponding segmentation image (scale bar: 400 µm). Inset (right) shows a zoom-in view on cortex ipsilateral to the stroke with individually segmented cells shown in random colors overlaid on the original PI image (scale bar: 100 µm). **f** Segmentation results for layer-specific neurons using Thy1-YFP (YFP). Coronal view of a Thy1-YFP stroke brain and its segmentation image (scale bar: 400 µm). Insets (right) show zoom-in views on the cortex contralateral to the stroke (orange box) and cortex ipsilateral to the stroke (blue box) (scale bars: 50 µm). **g** Zoom-in on YFP results with segmentation overlaid on raw images, and 3D rendering of YFP raw and segmented neurons. **h** 3D rendering of an original YFP volume with a 5-µm isotropic resolution (left) and examples of voxelized segmentation results (where the segmentation images are summarized at lower resolutions in the Allen space) at 25 µm in two- and three-dimensions (scale bar: 600 µm).

For an integrated analysis of multimodal data, precise image registration is essential, which is challenging because of stark differences in contrast across modalities, as well as tissue deformation after stroke and clearing. Indeed, registration of our cleared mouse stroke brains using available registration software yielded suboptimal results (Supplementary Fig. 1), prompting us to develop a workflow designed with both biologically and technically induced tissue deformation in mind. We developed and optimized fully automated registration workflows based on the deformable B-spline algorithm implemented in *ANTs*^33^ to accurately map MRI and CT, as well as CLARITY whole brain and sections to the ARA space (Fig. 2a–c, Supplementary Fig. 2; “Methods—Registration”). To ensure an accurate part-to-whole registration for CLARITY sections, we developed a recursive, similarity-based algorithm to search the corresponding whole-brain CLARITY volume and find the segment that corresponds best to the desired input section. The algorithm relied on a pyramid scheme using a sliding window with decreasing increments (“Methods—Registration, CLARITY sections’ registration”). We validated our registration workflows using manually placed landmarks (average of 30/mouse) on anatomical regions of interest (including striatum, hippocampus, thalamus, and cortex) from all modalities and obtained an average target registration error (TRE) for all 10 stroke mice of 130 ± 40 μm (mean ± standard deviation) and 418 ± 257 μm for CLARITY to ARA and in vivo MRI to ARA, respectively (Fig. 2d, Supplementary Fig. 2; “Methods—Registration and Segmentation validation”). The improvement in registration accuracy of the CLARITY data when employing deformable fields in addition to affine transformations demonstrate that the registration procedure accounts for the tissue deformations that arise during the clearing process (Fig. 2d). Similar registration fidelity was qualitatively found in suboptimal CLARITY data (e.g., due to noisy microscopic data and volumes with large intensity inhomogeneity; Supplementary Fig. 2, Supplementary Movie 2). Furthermore, our registration workflow demonstrated similar fidelity on other freely available sample clearing data generated with other clearing techniques, including iDISCO+ and CUBIC, as well as serial two-photon stacks (Supplementary Fig. 3).

While the transformed MRI maps such as relaxation or diffusion maps already contain parameter information in all (larger) ARA regions, CLARITY cellular features require segmentation for the extraction of quantitative metrics, such as cell number. We built optimized segmentation workflows that incorporate image pre-processing and morphological analysis algorithms implemented in *ImageJ* including 3D filters from the *3D Image Suite*^34^ and a 3D watershed-based algorithm from the *Morphological Suite*^35^. In addition, we developed an automated, fast (through parallel computation) feature extraction algorithm that computes 3D cellular features including count, density, and volume from the segmented images (“Methods—Segmentation”) (Fig. 2e–h, Supplementary Fig. 4). We also build tools to summarize the segmentation results per registered Allen labels and generate voxelized feature heat-maps, enabling detailed single subject and group analyses. We validated our segmentation protocols using manually labeled Thy1-YFP neurons as ground truth in the cortex from 3 independent counts for control mice (Supplementary Fig. 4), achieving a specificity of 95.8 ± 3.1% and a detection rate of 91.7 ± 6.9% (“Methods—Registration and segmentation validation”).

### Linking macroscopic with microscopic imaging of acute stroke

**Fig. 3:**
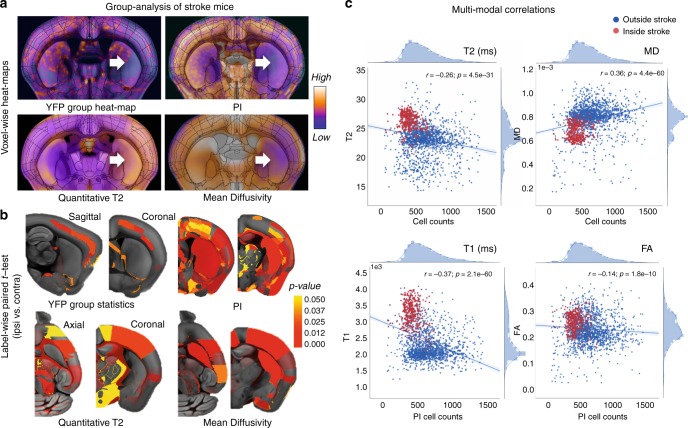
MRI measures correlate with CLARITY features in acute stroke. **a** Voxel-wise heat-maps (averaged across mice) depict the relative levels of yellow fluorescent protein (YFP), propidium iodide (PI), transverse relaxation time (T2), and mean diffusivity (MD) averaged over all 10 stroke mice, demonstrating acute changes in right-hemisphere stroke discerned by both CLARITY and MRI (white arrows). **b** Group-level statistical maps showing differences between ipsilateral and contralateral regions for Thy1-YFP, PI, T2, and MD. Color bar represents significance levels (label-wise *p* values) thresholded at alpha 0.05. **c** CLARITY–MRI correlations demonstrating a positive correlation of MD and a negative correlation of T1 with neuronal counts. Voxel-wise heat-map correlations of PI cell counts with quantitative MRI parameters (relaxometry: T2/T1, diffusion: MD, fractional anisotropy (FA)). Spearman’s rank correlations between PI cell counts and MRI parameters show a substantial decrease in MD values as well as an increase in quantitative T2 and T1 values in the stroke region (red) as compared to the unaffected brain regions (blue).

To determine imaging signatures of focal cellular changes after acute stroke (24 h post-stroke), we first compared group-level heat-maps for Thy1-expressing neuron (YFP) counts and cell nuclei (PI) with MRI measures (Fig. 3a). For MRI metrics, we quantified on relaxometry (T1 and T2) maps, which represent signal decay or relaxation times, and diffusion maps (mean diffusivity (MD) and fractional anisotropy (FA)), which characterize the rate of diffusion and degree of unidirectional restriction/hindrance in water movement. These voxelwise YFP and PI heat-maps showed cell loss in the caudoputamen (CP) and overlying sensory and motor cortex, with corresponding T2 and MD heat-maps showing in vivo changes to the same regions. To further assess inter-hemispheric differences within the stroke mice, a label-wise paired *t* test was calculated between ipsilesional (same side as stroke) and contralesional hemispheres in each ARA region for CLARITY cell counts and MRI measures (Fig. 3b). The pipeline accounts for the relatively lower resolution of MRI by grouping ARA labels to their “grand-parent labels” according to the ARA ontology hierarchy (e.g., the grand-parent label of layer 1 of the primary somatosensory nose area is the primary somatosensory area; Supplementary Fig. 1f). Both YFP and PI showed decreases in cell counts ipsilateral to the stroke in parts of the secondary somatosensory (*p* < 0.005 for YFP, *p* < 0.001 for PI using paired *t* test) and motor cortices (*p* < 0.01, *p* < 0.01). PI changes were both of greater magnitude and extent than YFP, spanning large cortical and subcortical areas such as the CP (*p* < 0.001 using paired *t* test), owing to more extensive PI labeling. The most prominent changes in T2 and MD were also in somatosensory (*p* < 0.01 for T2, *p* < 0.001 for MD using paired *t* test) and primary motor cortices (*p* < 0.01, *p* < 0.025 using paired *t* test). MD changes, known to be more sensitive to acute ischemic stroke, also encompassed subcortical regions such as the CP (*p* < 0.001), matching the changes on PI.

We performed voxelwise nonparametric Spearman’s rank correlations to quantify the relationship between CLARITY (PI) measurements and in vivo MRI (T1/T2/FA/MD) (Fig. 3c, red = inside the stroke core, blue = outside, voxel size = 25 µm). An inverse correlation was found for T2 (*r*_s_ = −0.26, *p* < 0.001 using Spearman’s rank correlation), T1 (*r*_s_ = −0.37, *p* < 0.001), and weakly with FA (*r*_s_ = −0.14, *p* < 0.001 using Spearman’s rank correlation), i.e., higher T2/T1/FA values were measured in regions of lower PI density associated with cell loss and cytotoxic edema. In contrast, MD, a measure of overall diffusivity in a voxel, correlated positively with PI density (*r*_s_ = 0.36, *p* < 0.001 using Spearman’s rank correlation), indicating restricted diffusion when cell counts were decreased. Ex vivo CT, after immersion in iodinated contrast, demonstrated a modest inverse correlation between quantitative Hounsfield units and PI density (*r*_s_ = −0.14, *p* < 0.01 using Spearman’s rank correlation), representing expected enhancement of the regions of infarction. The significant correlations between quantitative imaging and CLARITY-based histology, along with the registration and segmentation validation experiments, support the accurate translation captured by this resource from the microscopic to the macroscopic scale.

### Distant cellular changes in the acute phase after stroke

**Fig. 4:**
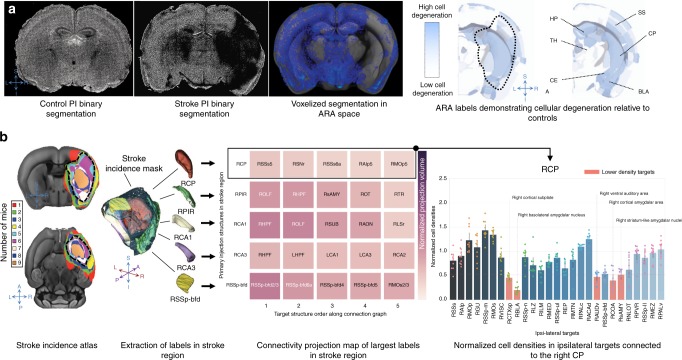
Observed cellular degeneration in remote region at the acute stage. **a** Visualization of cellular degeneration (relative to controls) due to stroke in ARA labels. From left to right: Binary PI segmentation of a control mouse (coronal view) showing similar nuclei counts across both hemispheres. Binary PI segmentation of a stroke mouse showing diminished cellularity in the infarct. The voxelized map (in blue, downsampled and registered to the ARA template in grayscale) also shows diminished cell density per voxel. Right: Two coronal slices of the ARA atlas with label intensity representing decreased cell density normalized to control mice. The dotted line approximately outlines the stroke region with a 50% incidence across the stroke cohort. Lower opacity corresponds to a decrease in cell density. SS: somatosensory areas, CP: caudoputamen, TH: thalamus, HP: hippocampus, CEA: central amygdalar nucleus, BLA: basolateral amygdalar nucleus, S–I: superior–inferior, R–L: right–left. **b** Investigation of cellular changes in the connected regions. Left: stroke incidence map demonstrating the incidence of an ARA voxel being present in the stroke cohort, with the black dashed line outlining an incidence of >50%. Center left: Rendering of the 50% stroke incidence mask depicting the largest five ARA labels in that mask (RCP: right caudoputamen, RPIR: right piriform area, RCA1: field CA1 of the right hippocampus, RCA3: field CA3 of the right hippocampus, RSSp-bfd: right primary somatosensory area, barrel field). Center right: projection targets of each of the largest five labels in the stroke mask (with each label as an injection sites), ranked by normalized projection volume (color bar). Right: The bar graphs represent PI cell density in our stroke mice normalized to controls. Salmon-colored bars indicate regions with cell densities lower than two standard deviations from the mean of all normalized regions. The strength of connectivity decreases from left to right and with increasing color brightness, with the most connected regions being darker and on the left. Individual data points for each target region are overlaid on its corresponding bar graph. Error bars represent 95% confidence intervals using 1000 bootstrap iterations.

Network disturbances outside the stroke core may reflect a fundamental tenet of the diaschisis theory that functional changes occur between focal ischemic lesions and intra- and inter-hemispheric connected regions^36,37^. To evaluate the potential effects of the primary stroke on regions distant from the stroke core, segmented PI stains were warped into the Allen atlas resolution by down-sampling and convolution (Fig. 4a, voxelized segmentation). We normalized cell density for each Allen label per mouse by the average cell density of control mice for that label. To visually depict cell degeneration, this normalized density was inverted (where brighter means more cell degeneration, Fig. 4a, right panel). A stroke mask was manually delineated in each mouse using the T2-weighted MR images. Since the stroke lesions are heterogeneous in volume, we created a stroke incidence map (i.e., the incidence of a voxel being present in the stroke lesion across mice in the cohort). This was achieved by warping the individual stroke masks to Allen space and summing them to produce this incidence map. The stroke mask with an incidence of >50% was chosen for the analysis of effects outside the stroke core (Fig. 4a, dotted black outline). Substantial cell degeneration was detected with PI within the stroke mask, most prominently in the CP, with a mean of 31% decreased density.

To interrogate cellular alterations in regions remote from but structurally connected to the infarct, we developed an automated tool to assess cellular features across neural networks (“Methods—Connectivity analysis”). Here we employed the Allen connectivity atlas^11^, which has been constructed from 469 injection experiments of adeno-associated virus (AAV) vectors from defined regions and cell types. By using a recursive hierarchical query of the injection site and its parent labels through interfacing with the Allen connectivity software, the pipeline automatically extracted the efferent targets from respective viral tracing experiments for the largest 50 ARA labels within the 50% incidence stroke mask (Fig. 4b, ≥5 affected mice, cyan mask with dotted black outline). The efferent targets for each injection were filtered to exclude children labels of the injection site as well as major parent labels (e.g., isocortex) and then ranked by strength of connection (projection volume normalized by volume in injected structure, with the top five targets/five labels shown in the middle panel of Fig. 4b). For each label in the stroke mask, we computed and plotted PI cell density (normalized to controls) in all of its connected targets (Fig. 4b right panel). We found numerous regions inside the ischemic lesion, including the CP, piriform area (PIR), and field CA1 of the hippocampus, with major projections to target regions with cellular alteration outside of the lesion (salmon-colored bars in Fig. 4b, Supplementary Fig. 5a). After careful exclusion of all targets with even minimal presence within the stroke mask of any mouse, several areas present as clearly affected and separate from the infarct (Supplementary Fig. 5b), demonstrating that cellular alteration is already observed in several areas highly connected to, but not within, the ischemic core. These changes at an early 24-h time point may be attributed to a vascular basis (i.e., microinfarcts not detectable by MRI or conversion of penumbra at the time of imaging to infarct at the time of sacrifice).

**Fig. 5:**
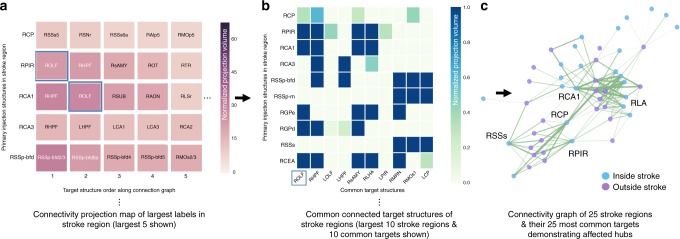
Examination of the involved networks and connected hubs in the stroke region. **a** Projection density map of the five largest regions in the stroke incidence mask (dashed line in Fig. 4b) as primary injection sites (*y*-axis) and their top five connectivity targets, ranked by connectivity strength (*x*-axis). Cell color in both the left and middle panels represents normalized projection volume between the primary injection (*y*-axis) and target structure (*x*-axis). **b** Matrix of the largest ten labels, as primary injection sites (*y*-axis), in the stroke area and their ten targets most common across all injection sites. **c** Snapshot of an interactive network graph of the largest 25 labels in the stroke mask and their 25 most common targets, highlighting example hubs (regions with a high degree of connectivity) inside and outside the stroke region. Blue nodes (circles representing brain regions) are regions inside the stroke and purple nodes are outside. Example hubs within the stroke: CP: caudoputamen, PIR: piriform area, SS: somatosensory areas.

To investigate not only the targets with cellular alteration but also their networks, the 50 most common targets for these 50 ARA labels within the stroke mask were also computed (top 10 shown in Fig. 5b). To interactively examine highly connected regions (hubs), the pipeline can display the connectivity information for these 50 stroke labels and their targets as a network graph (Fig. 5c) labeling region inside and outside the stroke or a connectogram (Supplementary Fig. 8) by ordering all regions according to their Allen hierarchy and depicting connections colored by injection site, weighted, and thresholded by the strength of connection (Top 25 shown in Fig. 5c). For these MCAO mice, this included several hubs involving striatal, somatosensory, and parahippocampal networks (Fig. 5c, Supplementary Fig. 8).

To further validate that our pipeline can detect cellular changes in connected regions secondary to connectivity to the stroke core, we employed histology data from 5 additional stroke mice that underwent MCAO and were sacrificed at day 15 post-stroke (“Methods—Histological validation”). 2D histological sections were examined at the thalamic–hippocampal level with: (1) CD68 to identify inflammatory cells (activated microglia and macrophages), (2) microtubule associated protein 2 (MAP2) to define areas of neuronal loss, and (3) 4′,6-diamidino-2-phenylindole (DAPI) for nuclear labeling (Supplementary Fig. 6a). We employed landmark-based registration to warp the Allen atlas labels to histology sections from a corresponding atlas section at the same depth (Supplementary Fig. 6b). Stroke masks of the infarct lesion were manually delineated on the histology sections using the available stains (Supplementary Fig. 6c). Our pipeline automatically identified regions outside of the stroke core, with significantly reduced ipsilesional MAP2 staining across the mice, including the retrosplenial area (RSP), arcuate hypothalamus nucleus, lateral hypothalamic area, and PIR–amygdalar area; which are not commonly part of the primary injury areas of this MCAO model (Supplementary Fig. 7a, b). These cellular changes in the top identified regions were further confirmed independently through visual inspection of the histological sections (Supplementary Fig. 7c). The top identified regions, several of which were revealed in our CLARITY-based stroke analysis, had large afferent projections from common stroke regions (Supplementary Fig. 7d), validating that our pipeline can identify cellular changes secondary to stroke in the highly connected regions.

### Automated resolution of axonal projection terminals

Although connectivity atlases based on discrete 2D high-throughput serial sections (such as the Allen atlas) can determine the projection pathway from an injection site, it is challenging to distinguish passing fibers from synaptic endpoints, making determination of fiber tract termination difficult^11^. In contrast, CLARITY-optimized viral tracing may enable the estimation of the number of terminating fibers within a brain structure in addition to the number of fibers passing through it through a 3D structure tensor analysis (STA) (“Methods—Structure tensor analysis and Projection terminals analysis”).

**Fig. 6:**
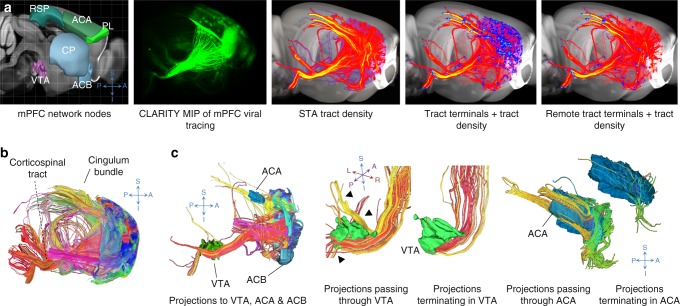
Computing streamline terminal zones and connectivity graphs from CLARITY viral tracing. **a** Generation of the structure tensor analysis (STA)-based tract density and remote tract terminal maps. First panel: Anatomical locations of several nodes of the medial prefrontal cortex (mPFC) network. Second panel: Maximum intensity projection of an original CLARITY volume with an AAV tracing injection at the mPFC. Third panel: Tract density maps (red) of STA-based streamlines from a CLARITY volume registered to the ARA. Fourth panel: Tract terminals map (blue dots) from the same specimen, both overlaid over the 25 µm ARA template and tract density map (red). Fifth panel: The same tract terminals map (blue dots) representing only terminals of long-range mPFC streamlines, with terminals near the injection site masked out, overlaid on the tract density map (red). **b** STA-based tractography of CLARITY viral tracing, colored by terminal location. **c** Depicting passing versus terminating mPFC tracts. mPFC streamlines to the ventral tegmental area (VTA), nucleus accumbens (ACB), and anterior cingulate area (ACA). Magnifications of passing versus terminating fibers in the VTA and ACA show more fibers terminate in the VTA than in the ACA.

We chose to study the mPFC, which is associated with several cognitive functions such as reward, fear, and addiction that may be served by unique networks, since the connectivity to this hub has been elucidated using a variety of data^39–41^. To obtain CLARITY-based tracts, we performed focal stereotactic injections of AAV expressing an axon-filling fluorescent protein^28^ in the right prelimbic area (PL) of the mPFC in three mice (“Methods—CAPTURE labeling”). Tract terminal maps from our CLARITY mPFC injections were generated by computing the number of STA-based streamlines ending in a voxel and the number of terminating streamlines summed per registered atlas label, resulting in a map of terminal zones (endpoints) (Fig. 6a, fourth panel; “Methods—Projection terminal analysis”). To interrogate projection terminals outside the immediate vicinity of the injection (i.e., avoiding proximal connections and/or tracer spill-over), the injection site and nearby structures were isolated through a connected components analysis, which labels neighborhoods of adjacent voxels that share the same set of intensity values. Masking the largest component (corresponding to the seed and nearby regions) leaves visible long-range tracts with terminals remote from the seed region (Fig. 6a, far right panel).

We filtered STA-based tractography results (Fig. 6b) by identifying tracts that pass through or terminate in several inclusion masks (Fig. 6c): (1) regions with many terminating fibers, for example, the nucleus accumbens (ACB) and the ventral tegmental area (VTA), as well as (2) regions with numerous passing fibers and relatively few terminating projections, such as the anterior cingulate area (ACA). In both cases, the terminals map showed the fibers connecting to the inclusion mask and filtered out long-range fibers passing through it (Fig. 6c, black arrowheads), suggesting an improved specificity for connectivity.

In order to directly compare the connectivity results between techniques, we graphically depicted Allen connectivity, CLARITY-passing projections, and CLARITY-terminating projections as interactive computational network graphs, with the injection site at the center and target structures as linked circular nodes (circles representing brain regions) (Supplementary Fig. 9a). CLARITY-passing projections were computed based on average tract density within an ARA region, while CLARITY terminal maps were computed based on the number of terminating fibers within the same ARA region. Larger nodes (circles) and thicker links (lines) both depicted stronger connections. We extended this visualization tool so that it can also be used to represent whole-brain connectivity, such as the entire Allen connectivity atlas (Supplementary Fig. 9c, Supplementary Movie 3).

We compared our tract density network graph derived from CLARITY streamlines without filtration of passing fibers (Supplementary Fig. 9a, middle panel), with the graph representing projection density from the PL area of the Allen connectivity atlas (Supplementary Fig. 9a, left panel). An overlap of 74% was found between the top 50 ranked targets by relative connectivity strength of the two methods (Supplementary Fig. 9b). We then compared to our graph based on projection terminals instead of passing fibers (Supplementary Fig. 9a, right panel), demonstrating an expected decrease in connectivity strength for the corpus callosum, which should not have terminating fibers. While this was manually corrected in the Allen connectivity atlas by removing signal from larger white matter fiber bundles, our terminal analysis automatically filters not only these larger bundles but all bundles of the passing fibers. For example, ACA (a structure without a known key functional role in the mPFC network) seems to be highly connected to the mPFC in the CLARITY graph without filtration of passing fibers (rank 3), but after terminal analysis, it showed a much lower connectivity strength (rank 54). Conversely, VTA is a known node important to the mPFC network^39^; however, its rank order is relatively low in Allen connectivity (rank 65). In the CLARITY network graph without filtration, the VTA is modestly higher (rank 39), but after terminal analysis it is much higher (rank 3). A similar effect was observed for ACB (Supplementary Fig. 9a). Similarly, while the CLARITY graph based on passing fibers suggested a much weaker connection between mPFC and ACB (rank 34), the graph based on terminal analysis revealed a higher number of fibers terminating in this key node (rank 12). Overall, the top projections based on CLARITY projection terminals match well some of the projections described in the reward and addiction networks^39–41^.

### Integrating dMRI, CLARITY, and the Allen connectivity atlas

**Fig. 7:**
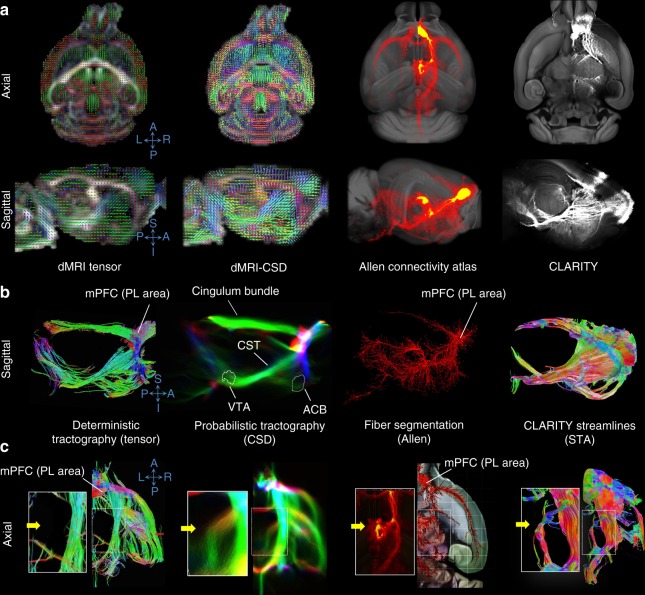
Examining prefrontal fiber projections using dMRI, the Allen connectivity atlas, and CLARITY viral tracing. **a** Raw and processed data across different connectivity scales (axial and sagittal views). Left panel: Ex vivo diffusion tensors (primary eigenvector for each voxel) overlaid on a fractional anisotropy (FA) map. Center left panel: Fiber orientation distribution computed using a constrained spherical deconvolution model (CSD, ref. ^44^) and overlaid on the FA map. Center right panel: Projection density map from a medial prefrontal cortex (mPFC) injection from the Allen connectivity atlas (red), overlaid on the 3D ARA template (grayscale). Right panel: Raw images from an example CLARITY viral tracing experiment with an mPFC injection, overlaid on a slice from the ARA template. A–P: anterior–posterior, R–L: right–left. **b** Tractography and fiber segmentation across different modalities from a sagittal view. The first two images show fiber tractography streamlines from diffusion MRI with different tracking techniques (deterministic tensor and probabilistic CSD), followed by a rendering of Allen connectivity, and lastly structural tensor analysis (STA) reconstructed streamlines from CLARITY in **a**. ACB: nucleus accumbens, CST: corticospinal tract, PL: prelimbic area of mPFC, VTA: ventral tegmental area. S–I: superior–inferior, R–L: right–left. **c** Resolving branching of fibers projecting from the prelimbic area (PL) to the mediodorsal nucleus of the thalamus (yellow arrow). Deterministic tensor tractography fails to capture this fiber branching, while probabilistic CSD tractography reproduced this projection that was confirmed in the Allen atlas and our CLARITY viral tracing data.

To further study the mPFC network at multiple scales in a common space, we produced connectivity maps by combining dMRI tractography, CLARITY-optimized viral injection experiments, and the Allen atlas of connectivity (Fig. 7a). At the macroscopic scale, tractography based on diffusion MRI provides an estimation of the most prominent fiber pathways, although validation of these tracts has not been fully achieved. At the microscopic scale, individual tracer injection experiments with 3D or 2D histology may offer a gold standard. However, they are limited in their ability to investigate numerous pathways simultaneously in a single mouse^13^. There is also considerable variance due to differences in tracer type, injection volume, and injection sites.

We relied on our ultra-high-resolution ex vivo dMRI scan to evaluate dMRI-based connectivity of the mPFC. We employed both (1) deterministic tractography using the diffusion tensor model, which computes only the primary water diffusion orientation in a given voxel^42^, and (2) probabilistic tractography as described before. Tracking parameters were kept constant between the tracking methods. To compare with CLARITY viral tracing, we reconstructed fiber tracts from CLARITY data through STA from our mPFC injections. Using our registration tools, we directly compared both dMRI and STA with the Allen mouse brain connectivity atlas^11^.

The diffusion model and tracking algorithm selected for analysis had a strong impact on correspondence between dMRI fiber tractography and both STA-CLARITY and the Allen connectivity atlas. The deterministic tensor and probabilistic constrained spherical deconvolution (CSD) fiber tracking demonstrated mPFC projections traveling to and crossing the ACB, as well as fibers joining the corticospinal tract (CST) and traveling caudally to the brain stem (Fig. 7b). Probabilistic CSD tracking, but not deterministic tensor, was able to resolve mPFC fiber bundles branching to the mediodorsal nucleus of the thalamus, which was observed in our CLARITY viral tracing and the Allen atlas (Fig. 7c, yellow arrow). Probabilistic CSD tract density showed a good agreement with projection density of the Allen brain atlas and the average of our segmented CLARITY viral tracings, with voxel-wise Spearman’s correlations of *r*_s_ = 0.31 (*p* < 0.0001) and *r*_s_ = 0.35 (*p* < 0.0001), respectively (Supplementary Fig. 10). Probabilistic CSD also showed a strong qualitative agreement with similar comparisons based on alternative probabilistic tracking algorithms described in the literature^43^ (Supplementary Fig. 11a). Deterministic CSD was intermediate between deterministic DTI and probabilistic CSD, capturing fiber bundles not seen on deterministic tensor tractography (Supplementary Fig. 11b, c) but with less extensive branching fibers than probabilistic CSD.

Comparing probabilistic CSD with both CLARITY and the Allen connectivity atlas, we found projections branching from the CST and crossing the striatum in both CLARITY and probabilistic CSD but not visible in the Allen connectivity atlas (Supplementary Fig. 10, white arrowheads). Overall, comparison of the average of our 3D viral tracings with the Allen connectivity atlas revealed a good correspondence (*r*_s_ = 0.46, *p* < 0.0001 for Spearman’s correlation), with several prominent bundles (corticospinal, cingulum) common between both tracing experiments (Supplementary Fig. 10—middle panel, Supplementary Fig. 11b). We also observed projections that were only visible in their entirety in the CLARITY experiments, specifically an axon bundle diverting at a sharp angle near the VTA from a laterally directed mPFC bundle, heading toward ventromedial thalamus (Supplementary Fig. 10, yellow arrowheads)^28^. This multi-scale approach can serve as a target toward validating and improving in vivo tracking algorithms and parameters.

### Thy1-YFP tract-level changes in the MCAO stroke model

We sought to integrate our tract-based tools with our stroke data in order to investigate stroke effects along major tracts in addition to remote regions of interest in our murine model. To define the tracts at high resolution, we performed an ultra-high-resolution ex vivo dMRI scan on one wild-type mouse. We then performed registrations between this dMRI scan and the same target Allen space where our stroke CLARITY data was warped. This enabled us to use our ex vivo dMRI acquisition to generate multiple fiber tracts within our stroke and control mice. Along these tracts, we quantified Thy1-YFP intensity in order to measure changes in cellular and fiber density (“Methods—Tract-level analysis”). An example region with tracts originating in the cortex and crossing the stroke is the mPFC.

**Fig. 8:**
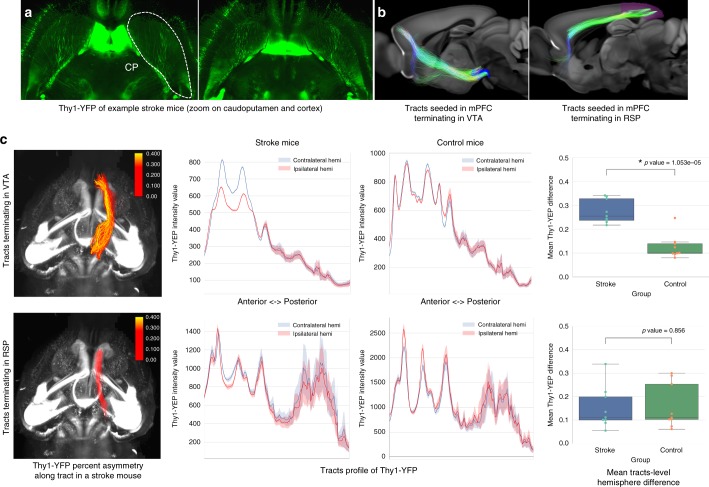
Thy1-YFP cellular modulations along dMRI-based tractography in acute stroke. **a** Thy1-YFP CLARITY axial images from a stroke mouse showing lower staining on the right hemisphere (right caudoputamen outlined). Images were intensity corrected for this figure (not for the analysis) to account for signal drop at the base of the olfactory bulb. **b** Two tracts were identified by probabilistic CSD tractography on our ultra-high resolution ex vivo dMRI dataset, seeding (starting from) the mPFC and targeting either VTA or RSP. Tracts were filtered to include only terminating fibers. **c** Cellular modulation along ipsilateral VTA but not RSP tracts in stroke mice compared to controls. Left: Track-based Thy1-YFP asymmetry depicted along these two tracts. Color scale represents percentage of asymmetry. Center: Tract-based Thy1-YFP intensity for the two tracts ipsilateral (red) and contralateral (blue) to the stroke, showing a decrease along the tract targeting the VTA but not the RSP. Right: Mean tract-level difference between contralateral and ipsilateral Thy1-YFP intensity, divided by the mean of contralateral Thy1-YFP intensity. *x*-axis of the graphs represents nodes (locations) along tracts going anterior (left) to posterior (right). Center line of box plot represents the median, bounds represent the first and third quantiles, and whiskers represent the lowest and highest datum within 1.5× the interquartile range of the lower and upper quantiles.

We thus used the mPFC as a seed region for tractography and selected two bundles: (1) mPFC tracts to the VTA (henceforth named VTA tracts), crossing the CP and joining the CST, which qualitatively intersected the stroke core and would be expected to show changes (Fig. 8a, b); and (2) mPFC tracts to the RSP (RSP tracts), joining the cingulum bundle, as control tracts which would not be expected to show changes (Fig. 8b). We performed probabilistic tractography using a CSD model, which models several fiber populations in each voxel (iFOD2 from MRtrix3)^44,45^. VTA tracts demonstrated significantly decreased Thy1-YFP values in the ipsilateral hemisphere (as compared to the contralateral hemisphere) in stroke but not in control mice (Fig. 8c top row, mean asymmetry for stroke = 27.1%, control = 11.8%, *p* ≤ 0.0001 using two-sample *t* test, example tract profiles for stroke and control mice in Supplementary Fig. 12). On a per mouse basis, 9/9 mice showed an asymmetry greater than the control average asymmetry. RSP tracts did not demonstrate a hemispheric difference in both groups (Fig. 8c bottom row, stroke = 14.9%, control = 15.7%, *p* = 0.856 using two-sample *t* test). On a per mouse basis, 3/9 mice showed an asymmetry greater than the control average asymmetry. Integrating dMRI with CLARITY and fiber quantification tools thus not only depicts changes to nodes through a label-based analysis but also changes along fiber tracts through a tract-based analysis.

It is unknown whether all tracts identified via probabilistic dMRI represent true axonal projections. To further investigate and validate these tract-level changes, we thus performed the same experiment based on tracts (extracted using STA) observed directly in higher-resolution viral tracing data (Supplementary Fig. 13a, b; “Methods—Tract-level analysis”). To obtain CLARITY-based tracts, we again used our mPFC injections. Similar to our dMRI findings, we observed significant changes in Thy1-YFP expression between the two cohorts along medial prefrontal tracts traversing the striatum (*p* = 0.00017) that would otherwise be difficult to detect with a region of interest (ROI)-based approach (Supplementary Fig. 13c, d, example tract profiles for stroke and control mice in Supplementary Fig. 13e). These findings highlight the potential of our resource to probe differences in features parametrized along fiber tracts between cohorts or perform unbiased automated projection mapping in defined activated states.

## Discussion

MIRACL is an automated pipeline that allows for multimodal interrogation of brain connectivity by coupling imaging of cleared volumes (using CLARITY or other clearing techniques) with MR/CT and the Allen atlas. We applied this general-purpose tool in an experimental stroke model and demonstrated group-wise alterations on both microscopic and macroscopic scales, with a strong correspondence between MRI and cellular changes in the region of ischemia. This analysis further demonstrated widespread acute cellular changes in regions strongly connected to the stroke area. In addition, we demonstrated histological changes along fiber tracts through the integration of dMRI and CLARITY. We improved the automated assessment of connectivity from CLARITY viral injections by computing maps of projection terminal zones, which more accurately match known patterns of connectivity. Finally, we integrated dMRI and CLARITY-optimized viral tracing with the Allen connectivity atlas in a standard reference frame. Through investigating the fiber bundles emanating from the mPFC across these three modalities and comparing them head-to-head, we found important differences between fiber tracing techniques, suggesting that CLARITY-optimized viral tracing may serve as a direct validation technique for diffusion tractography.

In our experimental stroke model, the artery occlusion leads to cell death primarily in the somatosensory cortex and striatum^46^. On the microscopic level, we detected the most prominent loss of Thy1-YFP neurons in layer 5 of the somatosensory cortex, a region delineated on T2-weighted images as part of the stroke lesion (Fig. 3c). CLARITY PI staining revealed that several areas connected to the stroke core, some remote to the lesion, were affected at the acute stage (Fig. 4b–d). At the macroscopic level, quantitative T2 values in the stroke region were substantially increased while MD values were decreased (Fig. 3c, d) due to a shift in water composition (e.g., the bound versus free water ratio), changes due to cytotoxic edema, and relative volume differences of intracellular and extracellular spaces^47^. The stroke mask used for CLARITY analysis was based on the hyperintense area in the T2-weighted MRI, which is expected to correspond to histology-based lesion at that time point^48,49^. Future studies will benefit from novel immunolabeling protocols applicable for the whole brain to include commonly used injury markers such as glial fibrillary acidic protein to visualize the formation of the glial scar around the ischemic tissue.

While our registration workflows produced accurate mapping both qualitatively and quantitatively (Supplementary Fig. 9), residual registration errors may still introduce variance. Our segmentation workflow included pre-processing steps and morphological operations to correct for possible inhomogeneities or artifacts in the PI stain. Parent labels of the Allen atlas were employed so that the connectivity analysis was robust toward residual inhomogeneities and artifacts within smaller labels. Future studies using MIRACL will integrate functional imaging and higher-resolution in vivo dMRI on a larger cohort of animals at both early and late time points post-stroke to further investigate the spatio-temporal effects in remotely connected regions and discern the nature of these cellular modulations.

Standard slice-based connectivity analyses are fundamentally limited: regional connectivity is often based solely on whether tracer or fiber tracts are visualized in a region (which necessarily includes fibers of passage), but it is more relevant to determine where neuronal fibers originate and synapse (terminate). Although larger white matter bundles have been manually removed as sources of passing fiber signal in the Allen connectivity atlas, there are many regions where fibers pass through gray matter without terminating. To address this issue of passing versus terminating fibers, we have demonstrated an automated technique to assess connectivity based on presumed projection terminals using CLARITY-optimized tracing, STA-based tractography, and atlas registration (Fig. 6, Supplementary Fig. 9). Incorporating projection terminals may be a more accurate method to assess connectivity strength as demonstrated for the mPFC. This algorithm is dependent on the accuracy of fiber orientation extraction and tractography stopping criteria, as well as intrinsic projection signal in the volume. We relied on the primary orientation of the structure tensors and the fiber assignment by continuous tracking (FACT) algorithm^50^ for our STA-based tractography. Future work modeling multiple fiber orientations with STA on tracing data should allow for more sophisticated models of fiber architecture. These models would enable the implementation of thresholds based on fiber orientation distributions for determination of tract terminals, in addition to the currently used tract curvature, providing more accurate estimates of tract endpoints. While our presented viral tracing experiments employed cytoplasmic enhanced green fluorescence protein (EGFP) AAV tracers, future work can further validate connectivity strength by direct electrophysiological studies or by employing a synaptic tracer like synaptophysin-EGFP-expressing AAV.

While other pipelines exist to compare across connectomes at different levels using graph theory and topology measures^51,52^ (Supplementary Table 1), our pipeline incorporates study-specific data with atlases to compare viral tracing or dMRI with atlas connectomes. It also enables extraction of connectivity from 3D cleared tissue through tact tracing with STA. Using our tools, we compared connectivity between modalities at varying resolutions by transforming dMRI, CLARITY-optimized viral tracing, and a connectivity atlas into a common space (Fig. 7, Supplementary Fig. 10). Although the main fiber bundles emanating from the mPFC were concordant among these modalities, several differences were present between deterministic tensor, probabilistic CSD dMRI tractography, and CLARITY tracing, with CSD having the best histological correspondence. Some tracts detected with dMRI (such as those branching from the cingulum bundle to the lateral posterior end of the RSP) were not observed in viral tracing experiments. This could represent retrograde fiber projections to the mPFC, possibly because dMRI is inherently bidirectional, whereas AAV viral injection-based CLARITY is efferent. Injection sites of our CLARITY experiments, and hence the STA-based tractography seed region, have also overlapped with regions of the mPFC other than PL. While STA has limitations, our STA maps accurately reflect connectivity known by entirely different methods (e.g., electrophysiological recording)^39^. Moreover, tract density images computed from CLARITY data were the result of 3D STA-based tractography and hence can produce more accurate representations of the fiber anatomy in all three dimensions, in contrast with density images acquired from the connectivity atlas that are generated through signal detection of 2D thin-section slices with a *z*-sampling of 100 µm^53^.

While label-based analysis is useful for identifying regional differences, it may not be optimal for changes along fiber tracts traversing specific paths in the brain. Investigating whole-brain histological changes across any chosen major tract of the mouse brain is one of the hallmarks of our tools, enabled by integrating dMRI, CLARITY, and fiber quantification techniques. We employed a high-resolution ex vivo MRI dataset to assess acute stroke changes along mPFC tracts terminating in VTA (joining the CST and crossing the striatum) and RSP (joining the cingulum bundle in the cortex). Through analysis of hemisphere asymmetry in Thy1-YFP labeling (image intensity) across these tracts, we found cellular and fiber density changes in the VTA tracts but not the control RSP tracts (when comparing stroke and control mice) (Fig. 8, Supplementary Fig 12). A more extensive analysis of tract-level changes is needed to determine the spatio-temporal effects of diaschisis on fiber tracts extending beyond the stroke core.

The presented resource enables high-fidelity 3D mapping of networks and cellular changes at the microscopic scale and their integration with in vivo connectivity maps and imaging metrics. Our application to stroke shows promise for discerning network alterations secondary to infarction at a cellular level across the brain. The improved modeling of connectivity based on CLARITY viral tracing enabled by our pipeline coupled with in vivo imaging has the potential to become a standard platform for assessing networks under experimental modulations. This tool is designed to harness information from large-scale projects and derive statistically relevant findings to answer specific questions about disease state and noninvasive biomarkers. MIRACL is readily applicable to a wide variety of neurological disorders, systems, and animal models and is adaptable to future evolutions of atlases and imaging methods.

## Methods

We describe below the animals and their manipulations; the in vivo and ex vivo imaging; CLARITY staining and microscopy; CAPTURE viral labeling; MRI, CT, and microscopy image processing; registration and segmentation; statistical analysis; feature extraction based on connectivity, dMRI tractography and CLARITY-STA tractography; and finally fiber terminal analysis.

### Animals and materials

All experiments were conducted in compliance with animal care laws and institutional guidelines, approved by the Stanford Institutional Animal Care and Use Committee, and in accordance with the guidelines from the NIH. We used *n* = 10 Thy1-YFP (line-H) mice, 8–10 weeks old (B6.Cg-Tg(Thy1-YFP)15Jrs/JC57BL/6J, Jackson Laboratory) and *n* = 10 wild-type littermates in the control group. All of the mice underwent light-sheet imaging of Thy1-YFP. Three of the 10 control mice underwent in vivo MRI and PI staining. Mice were housed under a 12:12 h light:dark cycle with food and water available ad libitum.

### Transient MCAO

Mice were kept anesthetized during surgery with 2–3% isoflurane in air. Body temperature, heart rate, and respiration were monitored every 15 min and kept in physiological range. The common carotid artery area was exposed and a silicon rubber-coated filament of size 7–0 (Doccol Corporation, Sharon, MA, USA) was inserted through the left internal carotid artery until the approximate branch of the left middle cerebral artery and left in place for 30 min to block blood flow. Subsequently, the suture was removed to allow reperfusion and wounds were sutured. To allow recovery from surgery, mice were administered with 0.01 mg/kg buprenorphine and 0.9% saline subcutaneously. Saline (25 µL/g body weight) was given subcutaneously 24 h later in order to help prevent dehydration due to reduced mobility.

### In vivo MRI

At 24 h after MCAO, mice were scanned on a 7-T animal MRI scanner (Agilent Technologies/Bruker) using a millipede coil. The animals were anesthetized with 2% isoflurane in air and fixed on a cradle to immobilize the head. Body temperature was maintained at 37 °C using constant air flow and the anesthetic concentrations were adjusted based on the respiratory rate.

A T2-weighted FSE (fast spin echo) sequence with echo time/repetition time (TE/TR) = 40/2500, a slice thickness = 0.5 mm, and 0.1172 × 0.1172 mm in-plane resolution was performed to get a high-resolution structural image of the brain. A T2 map was acquired using a 2D SE (spin echo) sequence with TEs = 12.1 ms, 24.3 ms, 36.4 ms, 48.5 ms, TR = 3500 ms, slice thickness = 1 mm, 0.5 mm gaps between slices, and 0.078 × 0.078 mm in-plane resolution. The T2 values were fitted using an in-house Matlab (MathWorks, Inc.) script. Keeping the same geometry, a T1 map was acquired with a FSE-IR (fast spin echo with inversion recovery) sequence, TE/TR = 7.2/5000, and inversion time = 200, 400, 800, 1600, 3200 ms. The T1 values were fitted using a Matlab script^54^. A higher-order shim (third) was performed to optimize magnetic field homogeneity in order to minimize imaging artifacts for the dMRI scan. The dMRI scan consisted of an EPI (echo planar imaging) sequence with bipolar diffusion gradients, TE/TR = 16.3/1200, 4 averages, 0.1172 × 0.1172 mm in-plane resolution, 1 mm slice thickness, 5 mm gap between adjacent slices, 3 *b* = 0 images, and 18 linear-independent diffusion directions with *b* = 1000 s/mm^2^.

### Ex vivo CT

A high-resolution ex vivo contrast-enhanced CT was acquired for all mice after immersion in an iodinated contrast agent (Omnipaque^®^, GE-HealthCare) on a MicroCAT II micro-CT scanner (Siemens Preclinical Solutions), using the following parameters: X-ray voltage 48 kV and anode current 200 µA, and a 0.6° rotation step throughout 198°, with a resulting isotropic voxel size of 18 µm.

### Ex vivo high-resolution MRI

A high-resolution ex vivo structural and diffusion MRI scan were performed of an excised control mouse brain (not part of the previous control cohort). The structural scan was a T1-weighted FLASH (fast low angle shot) sequence with TE/TR = 20/42.9, 8 averages, and 100 µm isotropic resolution. The diffusion scan had TE/TR = 28.8/500 ms, 200 µm isotropic resolution, 70 *b* = 0 scans, 150 directions with *b* = 1000 s/mm^2^, 230 directions with *b* = 2000 s/mm^2^, 270 directions with *b* = 4000 s/mm^2^, and 350 directions with *b* = 8000 s/mm^2^.

### Tissue clearing

CLARITY was performed as previously described^15,28^. Briefly, paraformaldehyde (PFA)-fixed brains were transferred into a CLARITY monomer solution consisting of 1% Acrylamide, 0.125% Bis-Acrylamide, 4% PFA, and 0.025% VA-044 initiator in 1× phosphate-buffered saline (PBS) for 3 days at 4 °C, polymerized by degassing and incubating at 37 °C for 3–4 h, and cleared passively in 4% Sodium Dodecyl Sulfate for 3 weeks at 37 °C in a 50-mL falcon tube. Samples were then washed with PBS + 0.1% Triton-X (PBST) for 2–3 days and placed in RapiClear (SunJin Labs) 12 h prior to imaging.

### Staining and microscopy

*Light-sheet imaging*: Whole-brain images were acquired with an Ultramicroscope II (Lavision Biotec). Samples were mounted to a custom 3D-printed holder using RapiClear Mounting Gel (SunJin Lab). They were securely mounted to the holder after mounting gel solidified (~5 min at 4 °C). Mounted samples were imaged inside an imaging chamber filled with 150 mL of RapiClear (reusable by periodical filtering). Samples were left in the imaging chamber for 20–40 min before imaging to allow the equilibrium of imaging solution. Brains were imaged using a ×2/0.5 NA objective at ×0.6 zoom. Multi-color imaging was enabled by setting filters to a supercontinuum white laser (NKT photonics). Samples were imaged with two light sheets (NA = 0.144) illuminating from both sides of the sample. *Z*-step was set to 5.16 µm (at ×0.6 zoom). Five horizontal focal points were set to each imaging plane for creating a homogeneous field of view. Following imaging, the samples were returned to PBST and sectioned at approximately 2–3 mm for confocal imaging with a razor blade.

*Confocal imaging*: Cleared tissues (2–3 mm coronal sections) were incubated in PI solution (Cell Signaling Technologies) for 2–3 days, returned to PBST, incubated in RapiClear CS for 1 day, and mounted using a Wilco dish. The tissues were then imaged using an Olympus FV1200 system equipped with a ×10 water-immersion objective (numerical aperture: 0.6; working distance: 3 mm; step size, 5 µm).

### CAPTURE labeling

Tissue clearing and whole-hemisphere light-sheet imaging were performed as described in the tissue-clearing section. Virus axonal labeling was performed according to the CAPTURE method^28^. Briefly, *n* = 3 wild-type C57BL/6 mice (from JAX) were injected with a 1 µL AAV8-CaMKIIa-EYFP-NRN in area PL within the right mPFC (AP = 2.0, *L* = 0.3, *V* = 2.5 mm). After injection, the mice were returned to their home cages for 4 weeks to allow the full expression of fluorophores. For light-sheet microscopy, all raw images were acquired as 16-bit TIFF files. The raw images were further processed by blind 3D deconvolution using AutoQuantX3 (Media Cybernetics) before STA.

### Whole-brain CLARITY image registration to ARA

The goal of the registration procedure is to have seamless mapping between MRI/CT, CLARITY, and ARA. This was achieved by computing whole-brain CLARITY to ARA transformations (CLARITY-ARA), and MRI to ARA transformations (MRI-ARA). Within CLARITY, stains of sections of the CLARITY volumes were registered to the whole-brain CLARITY, then easily to ARA. Similarly with MRI, all MR images were registered to the whole-brain T2-weighted images, which were themselves registered to the ARA.

To perform accurate mapping between CLARITY and the ARA, we developed specialized workflows optimized for multi-modal registration of clarified data, based on tools from *ANTs*^33^ (http://stnava.github.io/ANTs/). The workflows included preprocessing, brain extraction, intensity correction, orientation estimation, and registration initialization, as well as optimized similarity metrics and optimization and regularization parameters for the multi-stage, multi-resolution registrations. The following describes the preprocessing and registration protocols of whole-brain CLARITY to ARA templates.

To facilitate nonlinear registration of CLARITY to the ARA space, an autofluorescence channel was acquired, which provides detailed anatomical information and sufficient contrast, as well as a clear brain outline (Fig. 2a). The resultant transformation matrices and deformation fields were then applied to the remaining CLARITY channels (Fig. 2b). The CLARITY autofluorescence channel (594 nm) tiff data were first converted to an image volume of nifti format. During conversion, CLARITY data were downsampled by a factor of ×5. The input image was then intensity corrected using the nonparametric N4 bias field correction algorithm^55^ in order to eliminate non-uniformities (inhomogeneities) and shading artifacts. We then smoothed the image using a median filter with a radius of three voxels. The dataset was also masked using a series of thresholding, erosion, connected components analysis, and dilation steps to remove any outlines produced during microscopy not belonging to the brain. Finally, the processed input image was oriented to the “standard” orientation matching the ARA template to maximize similarity between the two volumes.

Image registration between the autofluorescence CLARITY volume and ARA template relied on intensity-based alignment. The ARA 25 µm template was chosen as the reference image for the registration steps and the ARA 25 µm labels were subsequently warped and up-sampled to CLARITY full-resolution, native space. The registration parameters were chosen after testing a large parameter space and qualitative assessment of the fidelity of the alignment for multiple datasets by visual inspection of overlaid images, utilizing the same parameters across all mouse brains. To visualize registration results, we constructed look-up tables (LUT) for use in the open-source *ITKsnap* (http://www.itksnap.org) and *Freeview (Freesurfer)* (https://surfer.nmr.mgh.harvard.edu) software, so that registered label information (name and id) is displayed where the user interactively moves the cursor (Supplementary Fig. 1d). The first registration step was an initial alignment using the *antsAffineInitializer* tool from *ANTs*^33^, with a search factor of 1 degree (search increments), a search around the principal axis of 1 radian, and a local optimization of 500 iterations run at each search point. The second registration step consisted of an intensity-based b-spline, three-stage registration with increasing degrees of freedom of their transformations, encompassing (a) a rigid 6 degrees of freedom (DOF), (b) an affine (12 DOF), and (c) a non-rigid (deformable) *b*-spline symmetric normalization (SyN) stage, each consisting of a multi-resolution approach with 4 levels. We employed the mutual information (MI) similarity metric for the rigid and affine stages, using a spline distance of 26, 32-bins for histogram sampling, a convergence tolerance of 1e−10, and Gaussian regularization of the deformation fields. We utilized cross-correlation (CC) for the deformable stage^56^, with a radius of 2 mm and a gradient step length of 0.1. The net product of the registration is a transformation that performs bidirectional warping of images to and from the CLARITY native space, to and from ARA templates, and labels.

### CLARITY sections’ registration

In order to register the 2–3 mm CLARITY sections (that comprise approximately quarter of the whole brain) to the ARA template, we first registered the YFP channel of each section to the whole-brain YFP then utilized the warping of the whole brain to the ARA. We developed a recursive search algorithm to search the CLARITY volume using a pyramid approach and find the segment that corresponds best to the desired input section. Specifically, the whole-brain image was cut into multiple segments (based on the input section size) and the mean squared difference was computed as a similarity measure between each of these digitally extracted segments and the input cut section. Then five rounds of recursive search were performed where different segments were extracted around the initial segment with the highest similarity. The extraction was performed using a sliding window with decreasing increments. The final extracted segment with highest similarity to the cut section was set to be 15% larger than the section to account for matching errors at the section boundaries. A standard *b*-spline registration with a rigid, affine, and deformable stages was subsequently run using the matched segment as a reference image. This was followed by warping the CLARITY sections (both YFP and PI) to the ARA template using the part-to-whole as well as CLARITY to ARA deformation fields. The resampling was performed in one step by combining all the respective transformations and deformation fields.

### In vivo and ex vivo imaging registration to ARA

All in vivo MRI sequences were registered, using an affine transformation, to the structural T2-weighted scan (0.1172 × 0.1172 × 0.5 mm resolution) to account for any movement during the scanning session. The reference T2-weighted sequence was in turn registered to the 25 µm ARA template to warp all quantitative maps to ARA space. A similar pre-processing and registration protocol to the CLARITY-ARA (described in previous section) was used for the MRI mapping. The main differences included a skull-stripping step for MRI, exclusion of the initial *antsAffineInitializer* registration step (due to the improved match of histogram statistics), a smaller number of histogram bins (8) for MI in the rigid step, and the use of the CC similarity metric in the affine registration step. Furthermore, a radius of 8 was used for CC in the multi-resolution, symmetric *b*-spline deformable stage. All in vivo MRI maps were co-registered to the highest-resolution structural MRI volume (in this case, the T2-weighted sequence) and warped to ARA space by employing deformation fields between the ARA 25 µm template and structural MRI data (Fig. 2c). The same steps were employed to register our ex vivo CT data.

### Segmentation and feature extraction

We developed 3D segmentation protocols for both types of data (channels) we acquired: a sparse neuronal segmentation protocol for YFP and a nuclear version for PI. The segmentation protocols were implemented as *Fiji* (*ImageJ*, https://fiji.sc) macros and included three main stages, image pre-processing to highlight features, a simple 3D segmentation to produce initial markers, and a Watershed marker-controlled segmentation to produce final, high-quality segmented images. Pre-processing consisted of background removal employing a rolling of radius 50, histogram normalization to first slice of the stack to deal with intensity inhomogeneity across slices, contrast enhancement, 3D median filtering to remove noise with a radius of 2 pixels^34^, computation of a local threshold using the Phansalkar method with a radius of 15 pixels, and finally computation of 3D local minima using a radius of 2 pixels. We then computed a simple 3D segmentation with thresholding to filter out large objects, as implemented in the *3D Image Suite*^34^. A marker-controlled 3D Watershed segmentation was subsequently employed, as implemented in the *Morphological Suite*^35^. The algorithm simulates flooding from input markers by transforming the input image to a topological surface. The median filtered image was used as the input image, the segmentation of the local minima as markers, and the local threshold as a mask image. The segmentation protocol was implemented such that its results do not rely heavily on input parameters like filtering radii. Parameter tuning for background removal, local thresholding, and filtering was performed by visual inspection between original images and segmented cells. For the PI channel, a similar protocol was employed for segmentation, without median filtering due to the much higher density of cells.

To summarize our segmentation results in ARA space and generate CLARITY heat-maps for MRI correlation, a down-sampling method was used to transform our full-resolution segmentation results into the 25- µm resolution space. The segmentation volumes (multi-label segmented cells or nuclei) were first convolved with a spherical kernel with a radius of 5 µm (with an area representing a voxel in ARA space). Then the number of labels (cells or nuclei) in each convolved sphere averaged as a voxel in the voxelized map. Feature extraction was performed on the voxelized map in 3D and was summarized by ARA region/label. Cell counts, cell densities, and volume statistics of the CLARITY stains were computed. Extraction of region properties was performed in *Python* using the *skimage* module; the *joblib* and *multiprocessing* modules were used for parallel computation. Parameter extraction was performed as well for imaging modalities, specifically in vivo diffusion and relaxometry MRI and ex vivo CT, averaged within warped ARA labels in native MRI and CT space.

### Registration and segmentation validation

To validate the accuracy of our CLARITY-ARA and MRI-ARA registration protocols landmarks were manually placed on anatomical regions by one author (Supplementary Fig. 1b). Landmarks were placed on native volumes of all modalities prior to any transformations. These landmarks were chosen so that they are clearly visible on both modalities and that they are well distributed within the volume (Supplementary Fig. 1b, c). An average of 30 landmarks were placed per volume for all ten stroke mice in the cohort. The landmarks were then transformed using the transformations and deformation fields computed during registration. A TRE was computed by averaging the root mean squared error between the sets of warped landmarks for all mice (Supplementary Fig. 1b, c). Landmark placement and TRE computation were performed in *3D Slicer* (https://www.slicer.org). To further test our pipeline, we utilized our registration protocol on a very noisy CLARITY dataset and volumes with large intensity inhomogeneity. The algorithm still produced adequate accuracy by visual assessment (Supplementary Fig. 1a). We also tested our registration protocol on different datasets, not included in our study cohort, and found it to produce very accurate alignment (Supplementary Fig. 1a). For segmentation validation, specificity was defined by the percentage of cells correctly detected: True positive/(True positive + False positive) and detection rate was defined as the percentage of ground-truth cells detected: True positive/(True positive + False negative). Numbers were means of three independent counts from cortical regions of control Thy1-YFP mice (Supplementary Fig. 4c).

### Statistical analysis

Heat-maps of all imaging parameters and CLARITY features were generated by computing the sum of squares across all registered images (mice) per modality in the ARA space. To assess within-modality effects, Student’s paired *t* tests (two sided) were performed between the ipsilateral and contralateral hemispheres of each modality and within each ARA label across all stroke mice. To visualize the results, the color (intensity value) of each ARA label represented the *p* value of its respective *t* test to reflect the significance of the difference between hemispheres within the label for a specific modality (Fig. 2c). For assessing multi-modal correlations between imaging and CLARITY, voxel-wise Spearman rank correlations were performed between the PI cell count heat-map and imaging parameters across all voxels in the imaging volume (Fig. 2d). For the CT–PI correlation, we normalized by the contralateral side and excluded the ventricles with very dense iodinated contrast. Similarly, voxel-wise Spearman correlations were performed to assess the correspondence between CSD tractography and the segmented signal projection maps from the CLARITY viral tracing and the Allen connectivity atlas of the efferent mPFC projections (Fig. 3, Supplementary Fig. 12a, b). The maps were smoothed prior to registration using a Gaussian kernel with a standard deviation of one voxel, to account for registration errors. No additional data replications were performed. Statistical analyses were performed in *Python* (v. 2.7) using the *scipy*, *statsmodels,* and *scikit-learn* modules.

### Connectivity analysis

We sought to determine the regions connected to the tissue affected by stroke to begin interrogation into network effects of infarct. The stroke regions were first manually delineated for all mice on our highest-resolution images, the in vivo T2-weighted images that clearly delineate stroke at this 24-h time point. The segmented masks were then warped, using the nearest neighbor interpolation, to ARA space by employing deformation fields from our MRI-ARA registration. To compute stroke incidence maps, which quantifies the number of times an ARA voxel is delineated as a stroke, the warped masks were binarized and added together in ARA space. The stroke mask with the most cortico-striatal overlap (dashed black outline in Fig. 4b) was chosen as ROI for the connectivity analysis. All the ARA labels within the chosen mask were extracted and sorted by volume. For the analysis presented in the figures, we limited the visualizations to the largest 50 labels within the ROI. A search was then implemented to check if an injection experiment in the Allen connectivity atlas^11^ was performed with each of the 50 labels as an injection site. The wild-type strain (C57BL/6J) was chosen to constrain the search. For each label without an injection experiment, its parent labels were searched until an injection experiment was found. A query was then performed using the Allen Brain Institute connectivity API (http://help.brain-map.org) to extract structural connectivity information for each injection site. Structural connectivity was sorted by normalized projection volume (projection volume normalized by volume in injection structure) at the target structure from the injection. In order to focus on more detailed mid-ontology-level structures, we excluded major labels with ontology graph depth <5 and graph order <6. Children labels of an injection site were removed from the target list. To summarize the connectivity of the stroke region, a projection density map was computed with data for every injection site (label) presented as a row and decreasing connectivity of target structures by normalized projection volume from left to right (Fig. 3b, center panel). Finally, to elucidate networks and connectivity trends of the stroke region we computed the most common targets of the 50 labels and generated a connectivity matrix, as well as a connectogram of injection sites within the ischemic lesion and their common targets (Fig. 4, Supplementary Fig. 8).

### Cellular degeneration along connectivity tree

In order to assess cellular degeneration in remote areas to the stroke region, computed cell densities for stroke mice (using parent labels of the Allen atlas) were first normalized in relation to the average cell density of the control mice (per label). The reciprocal was computed, and this reciprocal was overlaid on the ARA labels, with increasing opacity of the ARA labels corresponding to decreasing cell densities (Fig. 3a—right panel). After computing the connectivity analysis and projection density map of the stroke area, we wanted to determine the relationship between cellular degeneration and connectivity away from the ischemic core. We utilized the connectivity information of the stroke ROI extracted in the previous section, and for each of the target structures of each injection site (label) we computed its normalized cell density (normalized by the control mice). We then split the ipsilateral targets (and their cell densities), presented as blue bars in Fig. 4b and Supplementary Fig. 5a. A mean cell density was computed for the ipsilateral structures and each structure with a cell density lower than two standard deviations from the mean was highlighted (salmon-colored bars). Each of the target structures with lower cell density was automatically identified from the ARA ontology graph to emphasize remote regions with increased cell degeneration that are highly connected to the ischemic lesion. To further investigate cellular degeneration within connected targets outside the stroke region, we performed an additional analysis where only mice with strokes equal to or smaller than (in volume) the 50% incidence map were included. Target structures with overlap with a stroke mask of any mouse were excluded. Similar to the previous analysis, graphs of normalized cell density were computed for targets (outside the stroke mask) connected to regions within the stroke mask (Supplementary Fig. 5b).

### Histological validation

Mice were sacrificed on postnatal day 15 and perfused transcardially with ice-cold PBS followed by 3% PFA. Brains were removed and cryo-protected overnight in a 20% sucrose/3% PFA solution. After the brains sank to the bottom, they were frozen on dry ice and stored at −80 °C until sectioning. Thirty-micrometer-thick sections were cut using a cryostat and kept at −20 °C in an antifreeze solution (30% ethylene glycol and 30% glycerol in PBS). For staining with two primary antibodies, sections were processed with the following steps: first washed in PBS, next incubated with pre-heated 0.1 M sodium citrate at 60 °C for 20 min for antigen retrieval. After that, sections were transferred to a blocking solution (10% normal animal serum, 1% bovine serum albumin in 0.3% PBS-triton X) for 1 h and incubated in a solution of primary antibodies for CD68 (1:500, Abcam ab53444) and MAP2 (1:200, D5G1, Cell Signaling Technology, Danvers, MA, USA) diluted in the blocking solution overnight at 4 °C. The next day, sections were washed in 0.3% PBS-triton X and incubated with secondary antibodies (1:500, Alexa fluor 546, Invitrogen A11081 for CD68; and 1:500, Alexa fluor 488, Invitrogen A32731 for MAP2) diluted in the blocking solution at room temperature for 2 h. DAPI (1:2000) was added during the last 5 min of the secondary antibody incubation. Sections were then washed in PBS, mounted, and coverslipped. Infarct lesion area was identified by CD68-positive activated monocytes/macrophages. We confirmed that CD68-positive areas matched the MAP2-negative areas (neuronal loss) (Supplementary Fig. 6a). Images were captured using a CCD camera at ×10 magnification (AxioCam MRm; Carl Zeiss AB, Switzerland), the Axio Imager M2 (Carl Zeiss AB, Switzerland), and the MBF software Neurolucida (MBF Bioscience, Williston, VT, USA). Stroke lesion quantification analyses were performed on coronal sections at the thalamo-hippocampal level (range: 1.3 mm to 2.1 mm posterior to Bregma). For alignment of the original histology image and ARA, the corresponding ARA 25 µm image was selected and scaled to the histology pixel dimensions manually. Landmark-based 2D image registration was performed in 3D Slicer (v4.6.2, http://www.slicer.org) using the thin-plate spline algorithm, modified from Ito et al.^57^. A set of 20–30 landmarks (fiducials) were placed on the moving (ARA) and fixed (histology) image to allow precise registration (Supplementary Fig. 6b). The resulting transformation was applied to the ARA template and labels. Registered images (ARA, ARA labels, microscopy channels) were combined as a stack in ImageJ (v1.5k, https://imagej.nih.gov/ij/). A manually defined lesion mask drawn using the available stains to identify stroke-affected ARA regions for each mouse (Supplementary Fig. 6c). MAP2 expression was quantified in the registered ARA regions from histological sections and a paired *t* test was performed (between ipsilateral and contralateral hemispheres) for the top 15 regions with low ipsilateral MAP2 across the cohort (Supplementary Fig. 7b). Afferent connectivity for the regions with significantly lower MAP2 ipsilaterally was automatically extracted from the Allen connectivity atlas. Briefly, a query was performed using the Allen Brain Institute connectivity API (http://help.brain-map.org) to extract afferent structural connectivity information for each identified region from histology (regions were defined as “target” structures in the connectivity search). Structural connectivity was sorted by normalized projection volume (projection volume normalized by volume in injection structure) at the target structure from the injection. In order to focus on more detailed mid-ontology-level structures, we excluded major labels with ontology graph depth <5 and graph order <6. We focused on the afferent connectivity to the top five regions highlighting large projections from stroke regions (specifically from CP and CA1) (Supplementary Fig. 7d). These projections (emanating from CP and CA1) and the identified target regions from histology were rendered (visualized) in the Brain Explorer software (https://mouse.brain-map.org/static/brainexplorer).

### 3D structure tensor analysis

We adapted the classic texture analysis technique called STA, from 2D to 3D, to recover local fiber orientations and reconstruct computational models of fiber bundle trajectories from image intensity gradients^28^ (http://capture-clarity.org/).

For each voxel, the local fiber orientation was first estimated as the tertiary eigenvector (i.e., with the smallest eigenvalue) of a structure tensor^38,58–60^, which was defined as:

1 $$S_w\left( p \right) = {\int} {{\int} {{\int}_{\!\!\!\!R^3} {w\left( r \right)S_0\left( {p - r} \right){\rm{d}}r} } }$$

where *p* and *r* represent spatial locations, *w* is a Gaussian weighting function with standard deviation *σ*_*g*_, *S*_0_ is a symmetric second-moment matrix derived from image intensity gradients:

2 $$S_0\left( p \right) = \left[ {\begin{array}{*{20}{c}} {\left( {I_x\left( p \right)} \right)^2} & {I_x\left( p \right)I_y\left( p \right)} & {I_x\left( p \right)I_z\left( p \right)} \\ {I_y\left( p \right)I_x\left( p \right)} & {( {I_y\left( p \right)} )^2} & {I_y\left( p \right)I_x\left( p \right)} \\ {I_z\left( p \right)I_x\left( p \right)} & {I_z\left( p \right)I_y\left( p \right)} & {\left( {I_z\left( p \right)} \right)^2} \end{array}} \right]$$

where *I*_*x*_, *I*_*y*_, and *I*_*z*_ are the gradients of image volumes *I* along each of the *x*, *y*, and *z* axes (a marker of the edges of fiber tracts), computed by convolving *I* with three 3D first-order derivative of Gaussian filters of standard deviation *σ*_dog_^61^. Parameters *σ*_*g*_ and *σ*_dog_ need to be adjusted accordingly based on the signal-to-noise ratio and imaging resolution of CLARITY images. Structure tensors were computed using Matlab (MathWorks, Inc.).

A deterministic FACT tractography algorithm^50^ was then employed to propagate streamlines from a “seed” region through the recovered vector field of voxel-wise STA-derived fiber orientations and terminates if a streamline makes a sharp turn (angles larger than a prescribed threshold *α*_thresh_ = 35°) or extends outside of the masked brain region. In regions of low fluorescent staining, the structure–tensor orientation becomes noisy, resulting in sharp streamline turns, and a streamline cutoff via our threshold, thus the angular threshold effectively serves as an analog of an FA threshold for DTI tractography in order to terminate a streamline.

### Tract-level analysis

In order to assess tract-level effects of stroke on CLARITY, all the whole-brain Thy1-YFP data (nine stroke and nine control mice) were warped to the ex vivo dMRI space (utilizing combined mapping to Allen space and ex vivo MRI). We first warped all the whole-brain Thy1-YFP data (stroke and control mice) to the dMRI space. Thy1-YFP volumes (for both stroke and controls) were flipped left–right and warped again using the same deformations in order to assess hemisphere differences with the same fiber tracts. Seeding again at the PL region, we employed the iFOD2 probabilistic tractography algorithm to track two pathways: (1) mPFC streamlines to the VTA, crossing the CP and joining the CST, and (2) mPFC streamlines to the RSP, joining the cingulum bundle. Tracking parameters were the same for both tracts, with a 0.2 fiber orientation distribution (FOD) threshold, angle threshold of 35°, and a maximum number of 5000 streamlines. Thy1-YFP values were sampled along the two tracts for both groups (stroke and control) and both hemisphere (with left–right flipped images used to compute contralateral tract profiles). Tract profiles were generated by averaging values for every ten adjacent streamlines for mouse (and hemisphere). Mean Thy1-YFP hemisphere absolute percent difference was computed for each group (stroke and control) and a two-sample Student’s *t* test was performed to assess the significance between groups for each tract.

A similar tract-level analysis was performed using STA-based tracts from a CLARITY-optimized viral injection (described in the “CAPTURE labeling” section). The whole-brain Thy1-YFP data (stroke and control mice) were registered and warped to the CLARITY space. They were also flipped left–right and warped again using the same deformations in order to assess hemisphere differences with the same fiber tracts. We then extracted mPFC streamlines to the VTA, crossing the CP and joining the CST, by employing STA and seeding at the PL region (as described in the “3D structure tensor analysis” section). Similar to the tract-level dMRI analysis, tract profiles were computed for STA streamlines and a two-sample Student’s *t* test was performed to assess the significance between mean Thy1-YFP percent difference of both hemispheres for both groups (stroke and control).

### Projection terminal analysis

Subsequently to performing fiber reconstruction through STA of cleared virus injection data, tract terminal (end-points) maps were generated, by computing the number of terminating tracts per voxel. The viral tracer expression highlights the full extent of pathways in 3D, and signal intensity diminishes at projections terminals, which can be captured computationally. A tractography algorithm (analogous to tractography based on dMRI)^23^ was used to connect these local fiber orientations and generate streamlines. These 3D reconstructions graphically depict tracts between a starting region, or seed region (analogous to an injection site), and a target region, producing long-range fiber pathways. At the termination of a tract, diminished signal results in a loss of reliable local fiber orientation detected by STA maps. This leads to sudden transitions in the fiber orientation and sharp turns in streamlines that exceeded typical curvature thresholds, resulting in the termination of tracking.

We relied on tract coordinates and mapping tools as implemented in the *Mrtrix3* software (http://www.mrtrix.org). In addition, we employed warped ARA labels based on the CLARITY-ARA transformation to compute terminal zones per mid-ontology anatomical labels from mPFC injections. These labels were created at mid-ontology level to focus on detail structures in the connectivity analysis. The streamline tractography results were then filtered by major labels involved in the mPFC network based on tract density maps from our previous analysis, for example, the ABC, VTA, and ACA. For each label, two sets of streamlines were computed by using it as an inclusion mask, identifying both (a) passing fibers through the structure and (b) fibers terminating within the structure. To highlight the differences of computing connectivity based on the two approaches (passing and terminating fibers), we summarized the connectivity results as computational network graphs (Fig. 6, Supplementary Fig. 11). Summarized data from tract density and tract terminal maps (i.e., the number of terminals) per ARA labels were thresholded by connection strength and transformed to connectivity matrices for visualization. Connectivity values from terminal maps were scaled for a comparable visual representation to the tract density map. We employed network graphs from the *Python* client of the *Lightning* visualization framework (http://lightning-viz.org/). Data from a wild-type PL injection experiment of the Allen connectivity graph was extracted and compared to our CLARITY network graphs. The projection density map was summarized per labels, thresholded by connection strength, and transformed to a connectivity matrix for visualization. The employed framework enables the generation of interactive web visualizations (Supplementary Fig. 11).

### dMRI tractography and comparison to Allen connectivity

To warp the ARA labels for tractography seeding and connectivity analysis, the high-resolution, structural ex vivo T1 FLASH was registered to the ARA 25 µm template using the MRI-Allen registration module of the pipeline (described above in “Whole-brain CLARITY image registration to ARA”). A deformable registration step between the structural MRI and the S0 (isotropic) diffusion image was performed using *ANTs*^33^. Deformation fields from both registrations were employed to warp ARA labels to the ex vivo diffusion space in a single step. The PL was then thresholded to be used as a seed for all the following DTI experiments. The raw diffusion data were first corrected for eddy distortions by registering each diffusion direction to the b0 image, using the *eddy* tool^62^ from *FSL* image analysis suite (https://www.fmrib.ox.ac.uk/fsl). The diffusion tensors from the standard diffusion model were then computed. For CSD-based methods, a response function was estimated and then used a kernel to compute an FOD per voxel. We performed three tractography experiments each with a different tracking algorithm using *MRtrix3* (http://www.mrtrix.org). The number of output streamlines was set to 500,000 for all the experiments. The first tracking algorithm used was FACT^50^, a deterministic approach based on the diffusion tensors and principal eigenvectors. We chose an FA-stopping threshold of 0.2, a maximum angle between steps of 25 degrees, and a step size of 0.01 was used. The second method was *SDstream*^45^, a deterministic CSD-based algorithm that utilizes the FOD information for tractography. A FOD amplitude cutoff of 0.2 was chosen. The last tracking experiment employed a probabilistic algorithm (*iFOD2*) that integrates over the FODs to compute fiber streamlines^45^, for which we used a FOD cutoff of 0.2. To compute a projection density image from tractography streamlines, tract density imaging (TDI) was used to produce a map where every voxel value corresponds to the number of streamlines passing through the voxel^63^. A tract density image was generated for the probabilistic tractography results (*iFOD* method) on a higher-resolution grid (25 µm) to match the ARA resolution.

To compare our diffusion tractography to the Allen connectivity atlas, the TDI map was then warped to ARA space using the transformations and deformation fields of the diffusion to ARA registration performed earlier. We then utilized the Allen Brain Institute connectivity API to query and download a projection density image of an experiment with the PL as an injection site. The experiment with the highest injection site tracer volume (0.14 mm^3^) for the wild-type C57BL/6J mouse line was chosen (exp. # 157711748). The injection had an overlap of 53% with the PL and 42% with the infralimbic area, corresponding to similar injections in our CLARITY viral tracing experiments. To automate this procedure, we have added a function in the presented pipeline, whereby a user specifies the desired injection site (label) and the script outputs the projection density image, connected labels (by id and acronym), and a projection map (similar to those presented in Fig. 5) for that injection experiment.

### Reporting summary

Further information on research design is available in the Nature Research Reporting Summary linked to this article.

## Supplementary information

Supplementary Information

Description of Additional Supplementary Files

Supplementary Movie 1

Supplementary Movie 2

Supplementary Movie 3

Reporting Summary

## Data Availability

A subset of the datasets generated and analyzed during the current study will be released as examples with the pipeline, and more are available upon request.

## Code availability

We have made our pipeline open access for the community. It is freely available at: https://miracl.readthedocs.io. It is implemented in a modular fashion with many well-documented core modules (functions) that can be executed as command-line tools or graphical user interfaces (GUIs), including registration, connectivity analysis, segmentation, label operations, workflows, visualizations, and statistics (Supplementary Fig. 14). We compiled thorough documentation with help functions for all commands, which is attached as supplementary material (MIRACL documentation).
