# Supplementary material for: Multimodal image registration and connectivity analysis for integration of connectomic data from microscopy to MRI
Source: Nat Commun. 2019 Dec 3;10:5504. doi: 10.1038/s41467-019-13374-0 (PMC6890789; doi:10.1038/s41467-019-13374-0)
Supplement: Supplementary file 1 — Supplementary Information [file 41467_2019_13374_MOESM1_ESM.pdf]

Multimodal image registration and connectivity analysis for integration of connectomic data from microscopy to MRI

Goubran et al.

Supplementary Material

Supplementary Table 1. Comparison of analysis pipelines

Pipeline	Clearing	Connectivity (network) analysis	Tract-level analysis	Atlas registration	MRI-atlas registration	Segmentation	Graph theory analysis	Atlas	Connectivity tools	Registration tools	Segmentation tools
Presented pipeline (MIRACL)	CLARITY / CAPTURE	✓	✓	✓	✓	✓	-	Allen	in-house, Allen API, MRtrix, FSL	ANTs, in-house	in-house, Fiji
Schwanke 2019 (NeuroVIIsas)	-	✓	-	-	-	-	✓	Multiple	in-house, NEST, DCA	-	-
Doerr, 2017	FluoBABB	-	-	✓	✓	-	-	MRI atlas	-	FSL	-
Niedworok, 2016 (aMap)	- (serial histology)	-	-	✓	-	-	-	Allen	-	NiftyReg, in-house	-
Renier, 2016 (ClearMap)	iDISCO+	-	-	✓	-	✓	-	Allen	-	Elastix	in-house
Susaki, 2014	CUBIC	-	-	✓	-	✓	-	Waxholm	-	ANTs	in-house

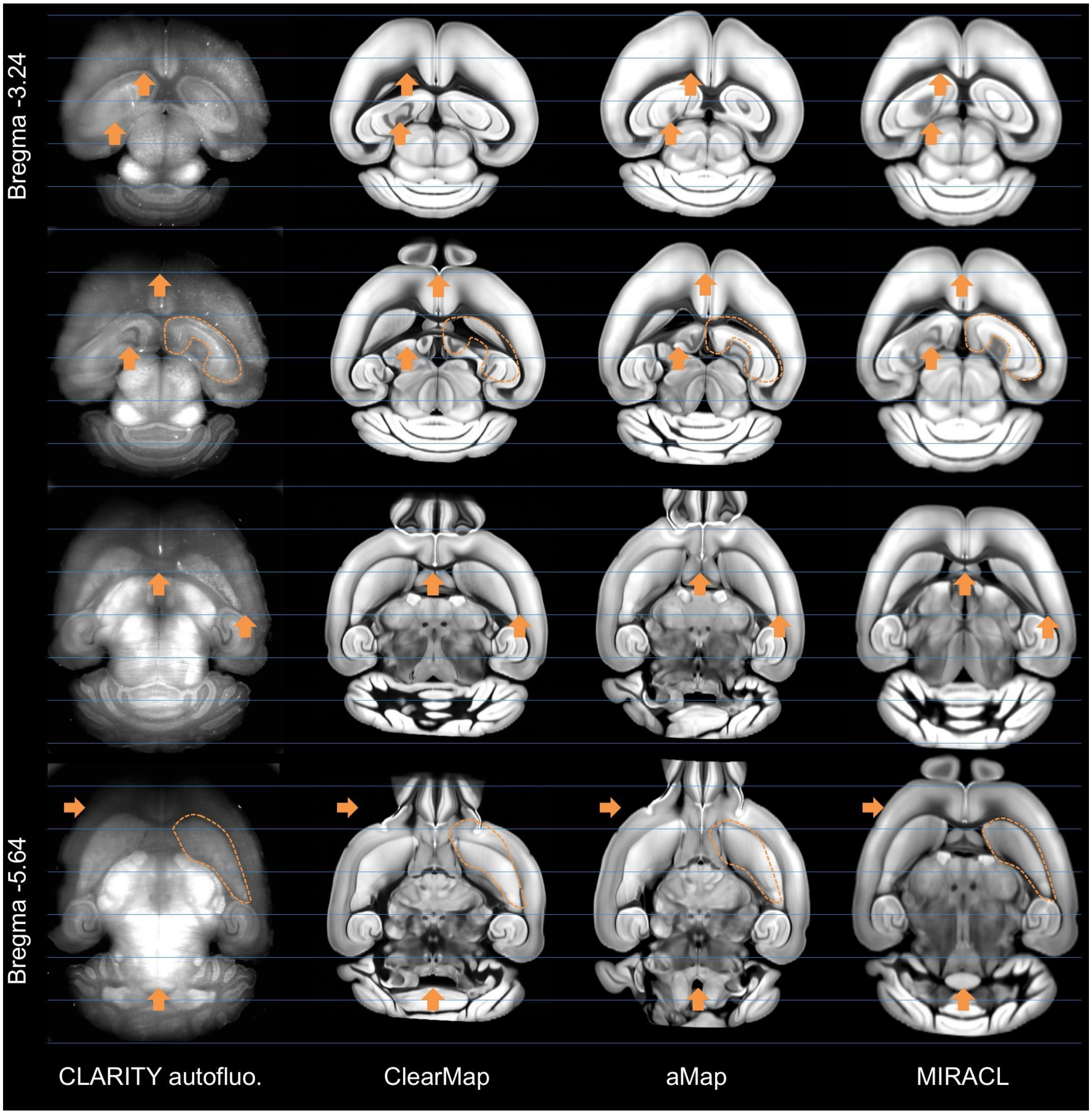

Supplementary Figure 1. Registration of a CLARITY stroke dataset across multiple pipelines.

Registration of a sample CLARITY dataset from our cohort (a stroke mouse) to the Allen atlas template across multiple pipelines with available registration software. The figure shows axial views of the Allen template warped to the CLARITY autofluorescence, showing the best alignment was achieved with our pipeline.

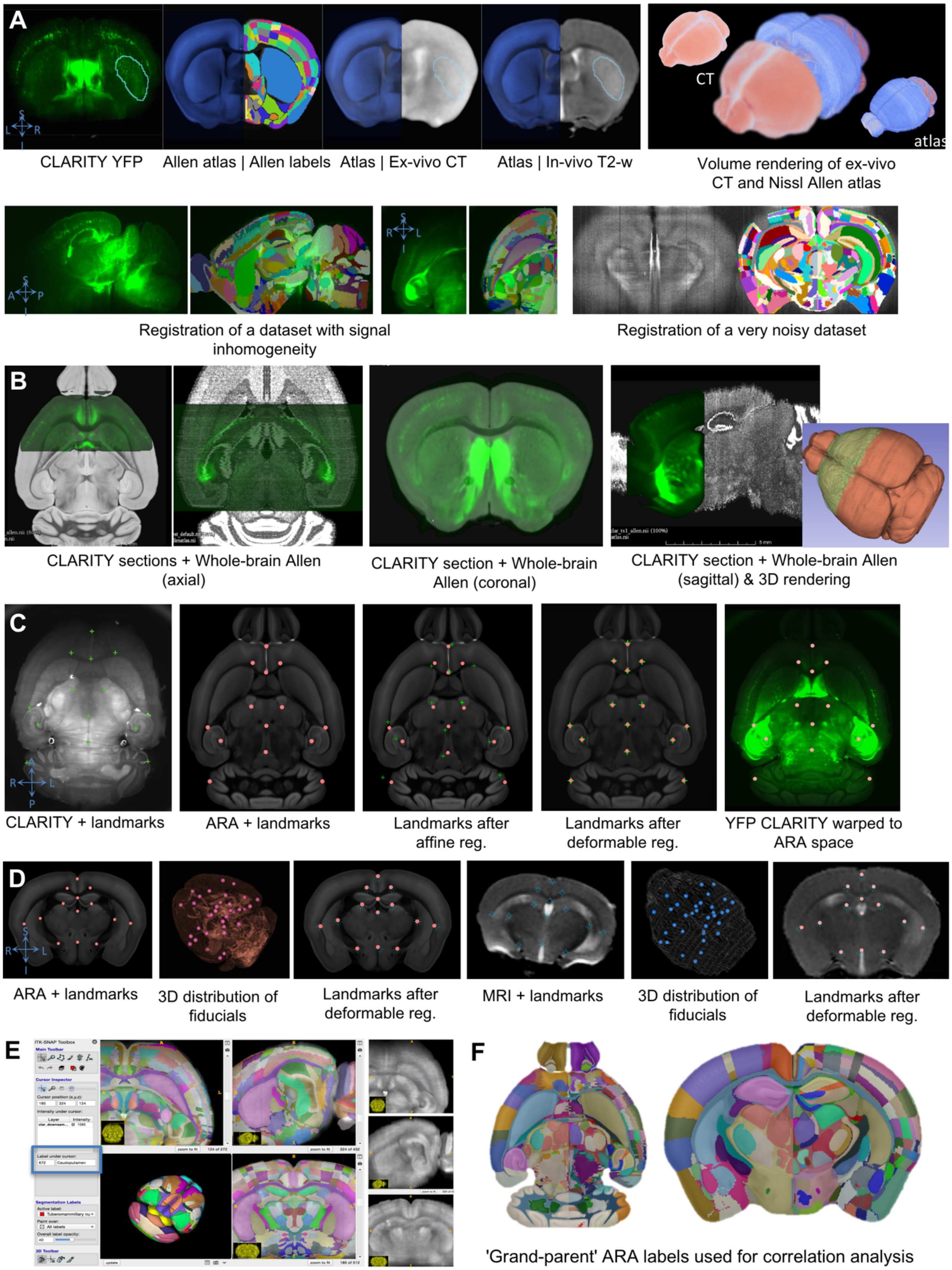
A
CT
atlas
CLARITY YFP
Allen atlas | Allen labels
Atlas | Ex-vivo CT
Atlas | In-vivo T2-w
Volume rendering of ex-vivo CT and Nissl Allen atlas
Registration of a dataset with signal inhomogeneity
Registration of a very noisy dataset
B
CLARITY sections + Whole-brain Allen (axial)
CLARITY section + Whole-brain Allen (coronal)
CLARITY section + Whole-brain Allen (sagittal) & 3D rendering
C
CLARITY + landmarks
ARA + landmarks
Landmarks after affine reg.
Landmarks after deformable reg.
YFP CLARITY warped to ARA space
D
ARA + landmarks
3D distribution of fiducials
Landmarks after deformable reg.
MRI + landmarks
3D distribution of fiducials
Landmarks after deformable reg.
E
F
'Grand-parent' ARA labels used for correlation analysis

Supplementary Figure 2. Registration results and target registration error computation for validation.

(A) Examples of MRI, CT and CLARITY registration to the ARA template. Top, left panel: Coronal sections of *in-vivo* T2-weighted MRI, *ex-vivo* CT and down-sampled CLARITY YFP of a stroke mouse registered to the ARA template. Images are ordered by increasing voxel resolution (from low to high). Top, right panel: Volume rendering of an *ex-vivo* CT of a stroke mouse (blue) and the ARA template (orange) in the ARA space, demonstrating the quality of alignment between the two modalities. Bottom panel: Registration results of difficult CLARITY datasets, with signal inhomogeneity (left) or noise corruption (right).

(B) Example of a CLARITY YFP sections registered to the whole-brain Allen atlas, demonstrating registration fidelity in the axial, coronal and sagittal planes, and a 3D surface rendering of the Allen volume with the registered the CLARITY section.

(C) Validation of CLARITY-ARA registration accuracy using manual landmarks. The MIRACL pipeline was used to create a mapping the CLARITY data to Allen space using affine transforms and non-rigid deformation fields. To inspect the fidelity of this warping, landmarks were manually placed in anatomical areas on native image spaces before transformation of both modalities (CLARITY and ARA template). The landmarks on the CLARITY images were then transformed by the MIRACL warping transformation to Allen space. Root mean squared error (RMSE) was computed between warped CLARITY landmarks and native ARA landmarks to compute target registration error (TRE).

(D) Validation of MRI-ARA registration accuracy using manual landmarks. Different landmarks were used compared to B because of differences in conspicuity of comparable structures. A similar procedure was employed to compute TRE between MRI and ARA.

(E) Visualization of CLARITY registration results by overlaying ARA labels on the downsampled CLARITY reference channel in the ITKsnap open-source software. A customized look-up table (LUT) was created so that label information (name and id) are displayed where the user interactively moves the cursor (blue box).

(F) 'Grand-parent' atlas generated for the voxel-based and statistical analysis of lower-resolution imaging modalities (MRI/CT). The atlas was created based on the Allen atlas ontology by grouping parent labels following the anatomical hierarchy.

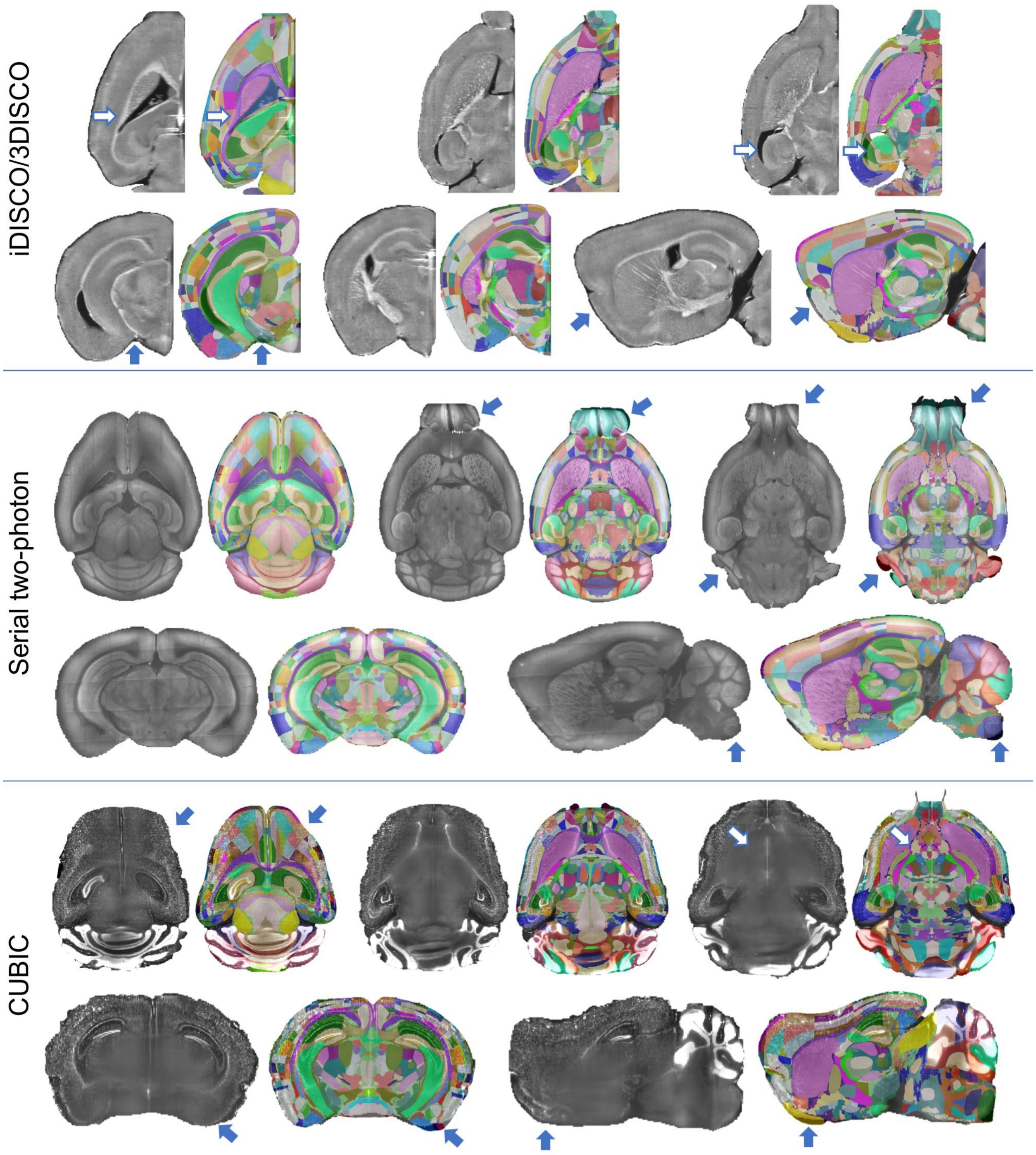

Supplementary Figure 3. Application of our registration workflow on data from different clearing and histological techniques

Results of registration to the Allen atlas of freely-available sample clearing data (other than CLARITY) including iDISCO+ (top row), serial two-photon stacks (middle row) and CUBIC (bottom row), showing high-quality registration across different techniques. The original clearing data are shown in axial, coronal and sagittal orientations with the overlaid warped Allen labels. Blue and white arrows show minor miss-registrations.

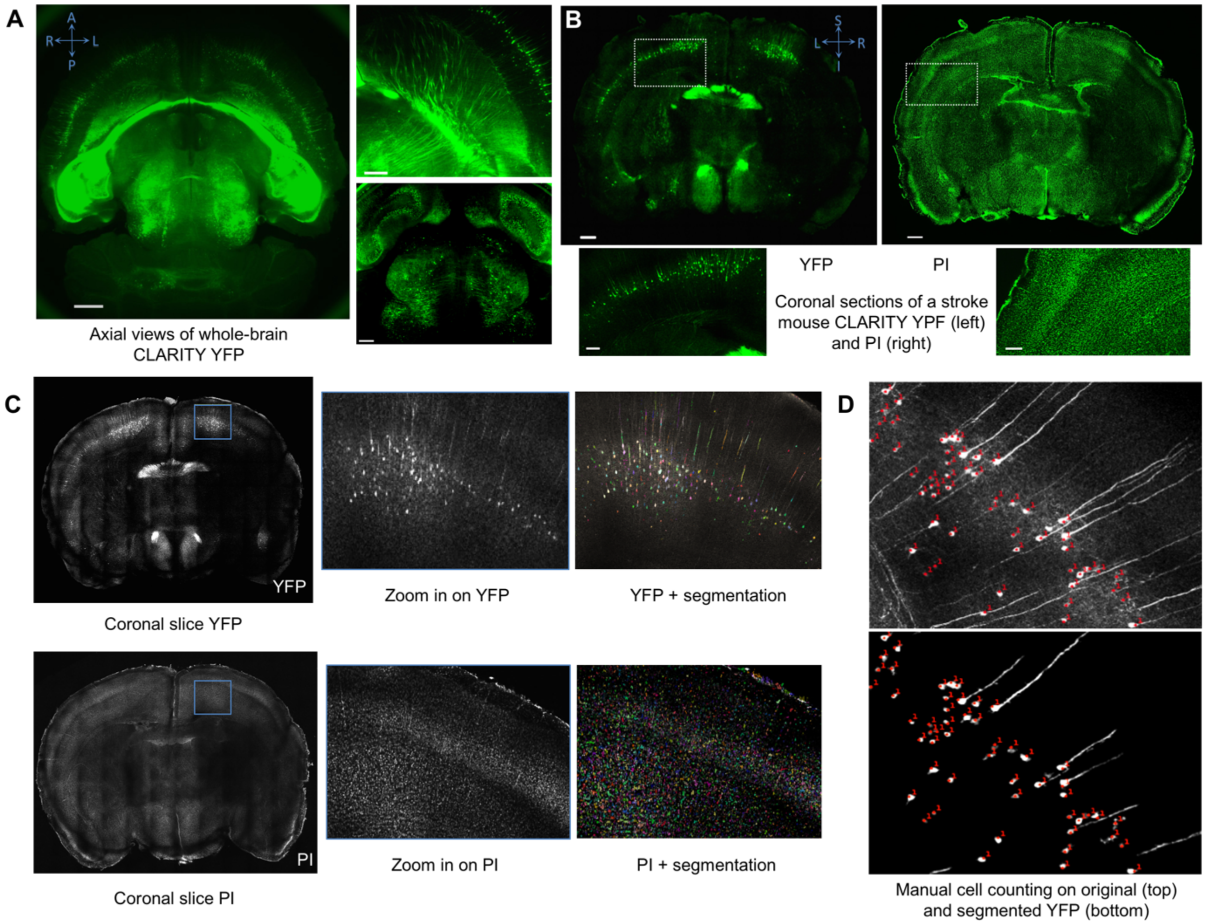

Supplementary Figure 4. Segmentation results and validation of the segmentation algorithm.

(A) Axial views of raw CLARITY YFP whole-brain data on a section without stroke. Scale bars: 600 (left), 300 (top), 200 (bottom) μm.

(B) Coronal views of YFP and PI CLARITY sections on a section with a stroke. Insets show zoomed-in windows on cortical neurons and nuclei. Scale bars: 400 (top), 100 (bottom) μm.

(C) Segmentation results of YFP and PI CLARITY. Top panel (blue boxes): Coronal zoom-in of the YFP and PI channels in grayscale. Bottom panel: Segmentation outputs for both channels, showing segmented neurons (YFP) and nuclei (PI) colored randomly and overlaid on the original grayscale image (with 50% transparency).

(D) Manual cell counting (labeled in blue) in the cortex for segmentation validation (quantification of specificity and detection rate) of the YFP channel. Counting was performed based on 3 independent trials for control mice.

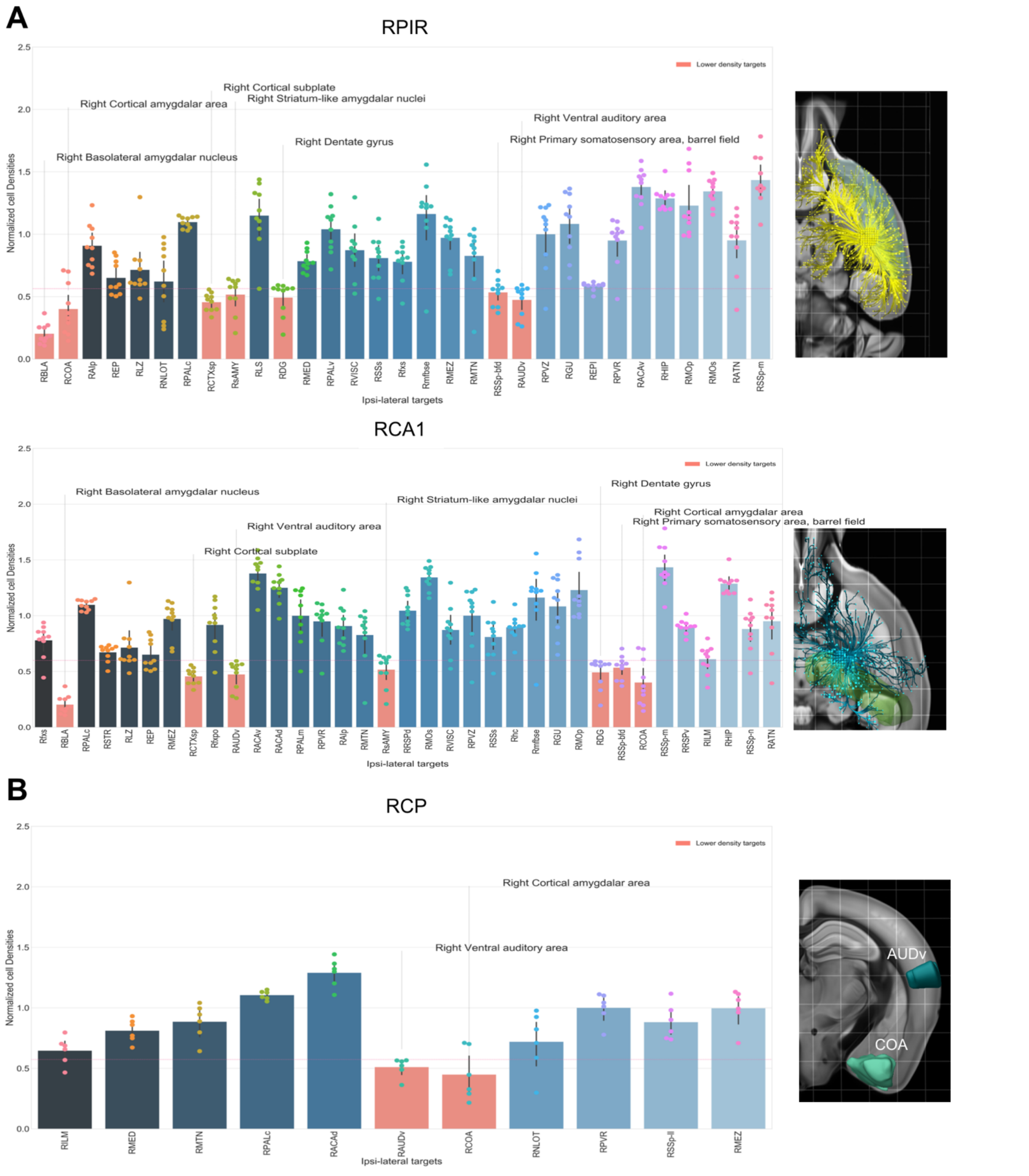

Supplementary Figure 5. PI cell densities along connectivity graphs of regions within the ischemic stroke.

(A) Normalized cell densities for ipsilateral targets connected to regions within the stroke mask (PIR = piriform area, CA1 = the CA1 field of the hippocampus, presented in **Fig 3).** Degree of connectivity (normalized projection volume) decreases from left to right and by color brightness, with the most connected

targets being dark blue and on the left of the graphs. Salmon-colored bars highlight targets with normalized lower cell densities (lower than two standard deviations from the mean). Individual data points for each target region are overlaid on its corresponding bar graph. Error bars represent 95% confidence intervals using 1000 bootstrap iterations. Right images demonstrate axial views of the region's ipsilateral connectivity, generated using *BrainExplorer 2* (Allen brain institute).
(B) Normalized cell densities for connected targets to the caudoputamen (CP), excluding targets with any presence whatsoever within the stroke mask of any mouse. The bars demonstrate that cellular alteration is observed in several targets highly connected, to but not within, the ischemic core. Right image demonstrates a coronal view of the anatomical location of the affected regions (AUDv = ventral auditory area, COA = cortical amygdalar area).

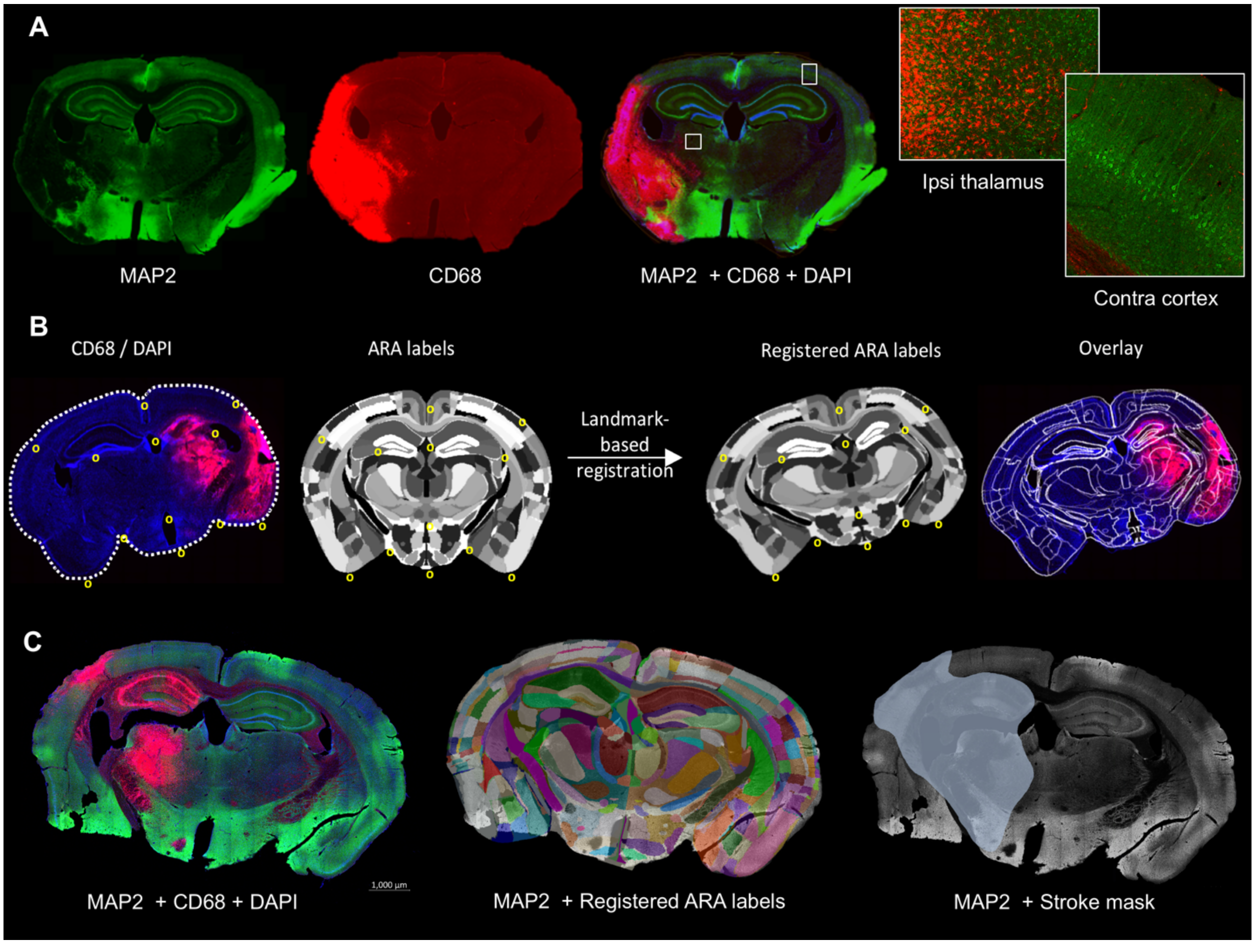

Supplementary Figure 6. Data and methods for histological validation.

(A) Examples of immunofluorescence stains MAP2 (green, absence of which indicates neuronal loss), CD68 (red, presence of which indicates inflammatory cells) and the overlay with DAPI (blue, presence of which indicates cell nuclei). Zoom-in of MAP2 and CD68 in the thalamus ipsilesional (ipsi) to the stroke and contralateral (contra) cortex, respectively.

(B) Registration of the Allen reference atlas (ARA) labels to the histology sections using manual landmark-based registration, to warp the Allen atlas labels to histology sections from a corresponding atlas section at the same depth.

(C) Example of the composed stains in another mouse along with the registered ARA labels and the manually delineated stroke region using available stains overlaid on a MAP2 stain.

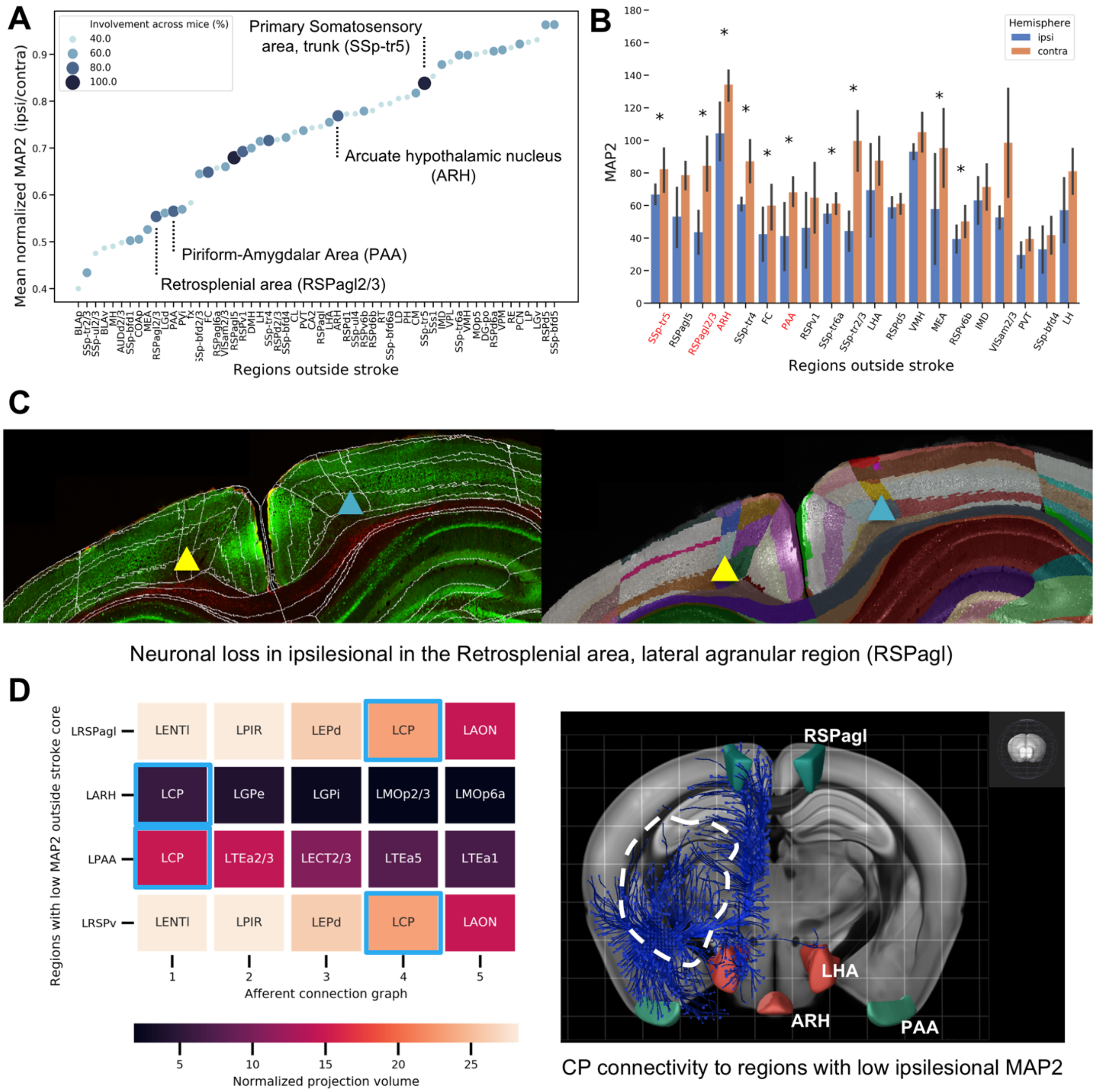

Supplementary Figure 7. Cellular degeneration in regions connected to the stroke core.

(A) Mean normalized MAP2 expression (ipsilateral over contralateral hemisphere) in regions outside the stroke core across mice. Regions were ranked by normalized MAP2 from lower (left) to higher (right). Circle size represents region involvement across the cohort (1/5, 2/5 ... or 5/5). Regions with the highest involvement are highlighted including: Retrosplenial area (RSPagl2/3), Arcuate Hypothalamus nucleus (ARH), Primary somatosensory area, trunk layer 5 (SSp-tr5) and Piriform-Amygdalar Area (PAA); which are not commonly part of the primary injury areas of this MCAO model.

(B) Ipsilateral and contralateral MAP2 expression in the top 15 regions ranked by mouse involvement. Asterisk represents significance in a paired t-test of region-wise ipsilateral and contralateral MAP2 expression. Regions highlighted in (A) are labelled in red. Error bars represent 95% confidence intervals using 1000 bootstrap

iterations.
(C) Example using traditional section-based histology of neuronal loss on MAP2 immunofluorescence stain in the ipsilesional Retrosplenial area (yellow arrows) as compared to the contralateral (blue arrows) hemisphere.
(D) Afferent connectivity to the affected regions from within the stroke core in the striatum (Left Caudoputamen - LCP). Left: Connectivity matrix of afferent connections to the top 4 regions with significantly reduced ipsilesional MAP2 outside the stroke core, ranked by normalized projection volume from left to right. Blue boxes represent the left CP region within the stroke core. Right: Rendering of CP projections (blue) to regions with low ipsilesional MAP2 from (A). Dotted white line depicts approximate stroke lesion border. Blue lines overlying this dotted white line projects outside the stroke core through-plane. Rendering generated using *BrainExplorer 2* (https://connectivity.brain-map.org/projection, Allen brain institute).

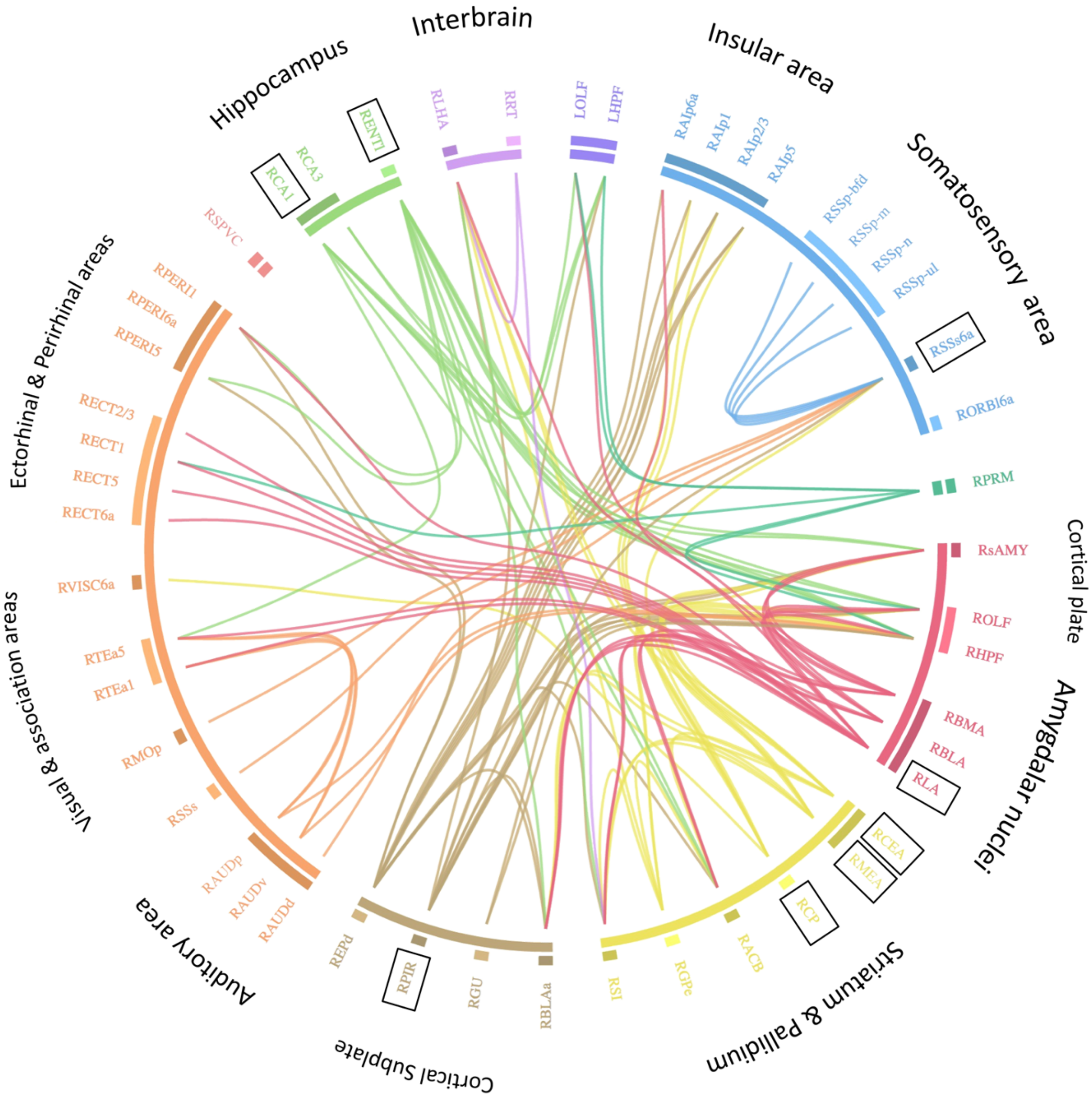

Supplementary Figure 8. Connectogram of 25 stroke regions and their 25 most common targets from healthy mice data

Snapshot of an interactive connectogram of the same labels and their 25 common targets. Black boxes indicate hubs in the stroke region. Labels are grouped according to the ARA hierarchal ontology graph into distinct functional areas, and colored corresponding to different brain areas. Only connections with normalized projection volume above 0.01 are shown.

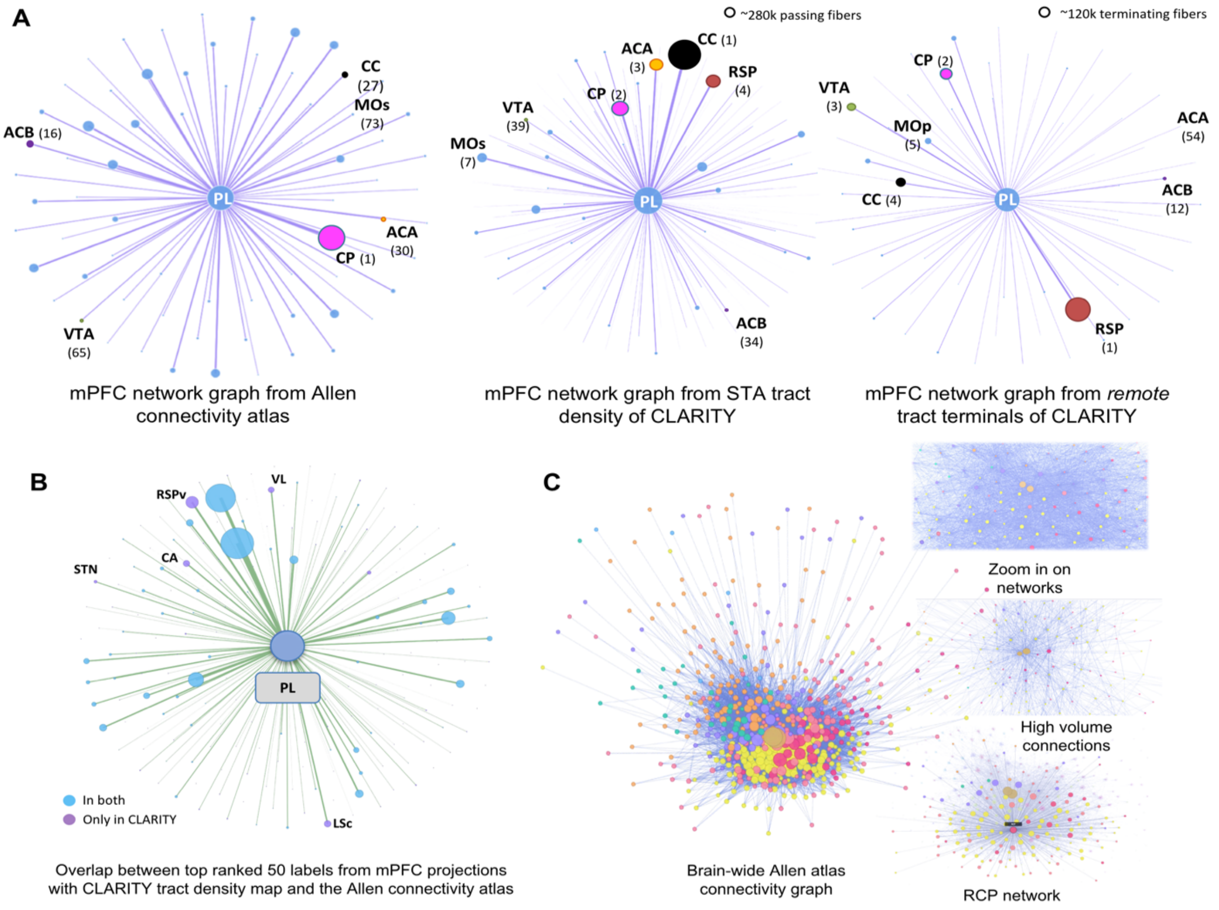

Supplementary Figure 9. Network graph comparison to the Allen connectivity atlas and visualization of brain-wide connections.

(A) Connectivity graphs of the mPFC network. The major structures in each graph are color coded and highlighted with rank order shown in parentheses. Larger node area and thicker links both represent stronger connections. Left: Network graph computed from the projection density of a PL injection from the Allen connectivity atlas. Middle: Graph computed from the STA tract density map of CLARITY, including both passing and terminating fibers. Right: Graph computed from the remote terminals map of CLARITY, including only terminating instead of passing fibers. While connectivity based on the STA tract density (passing fibers) suggested a much stronger connection between mPFC and ACA (a structure without a known key functional role in the mPFC network, rank 3) than VTA (a known node important to the mPFC network , rank 65), terminal analysis revealed a higher number of fibers terminating in the VTA. A similar effect was observed for ACB (rank 34 based on passing fibers vs. rank 12 based on terminal analysis). The relative prominence of RSP can be ascribed to injection of the adjacent cortex tracing back along the cingulum. PL, Prelimbic area; CC, Corpus callosum; CP, Caudoputamen; MOp, Primary motor area; MOs, Secondary motor area; RSP, Retrosplenial area.

(B) Overlap between the top ranked 50 structures (ARA labels) connected to the mPFC (PL area) from tract density maps of STA-based CLARITY streamlines, and a projection density map from the Allen connectivity atlas. Light blue circles (target structures) represent common structures in both connectivity graphs. Purple circles represent structures present only in the top 50 labels of the CLARITY tract density map. Connectivity strength, represented by node size and line width, is for STA-based CLARITY projection density.

(C) Whole-brain connectivity graph, based on all injection experiments from the Allen connectivity atlas data,

thresholded by connectivity strength. Insets highlight major hubs and connections as well as the Caudoputamen (RCP) network.

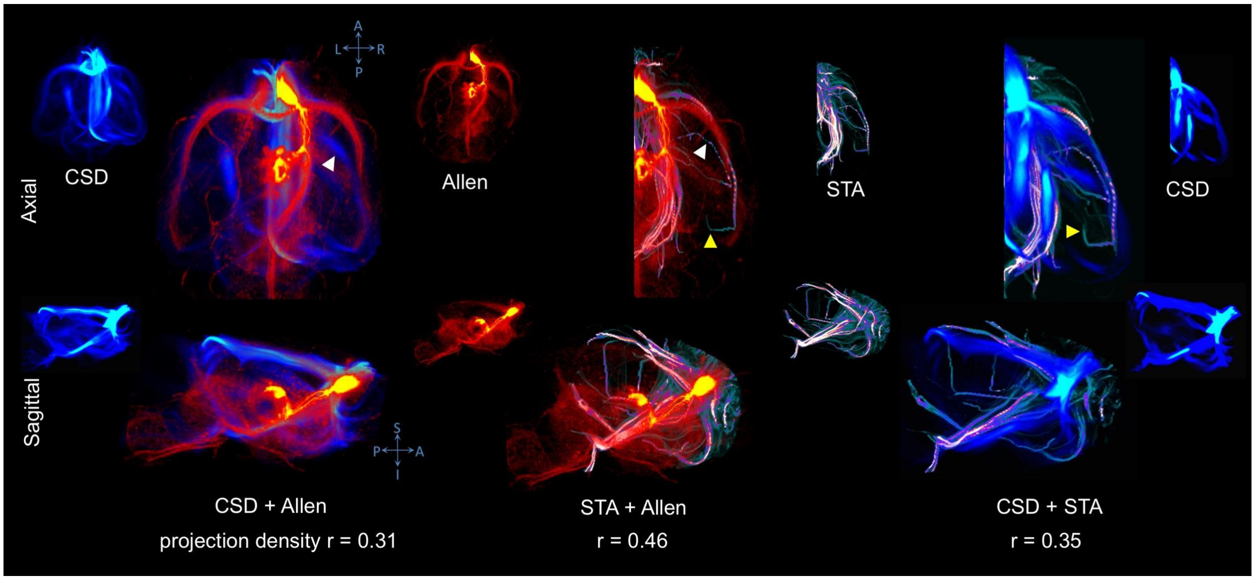

Supplementary Figure 10. Comparison of prelimbic area projection density between dMRI, Allen connectivity atlas and CLARITY.

Projection density images of the probabilistic diffusion CSD streamlines and CLARITY STA-based streamlines were both warped to ARA space. A strong agreement is observed between all three modalities. We also observed projections that were only visible in the CLARITY experiments, (yellow arrowheads), as well as projections observed in both CLARITY and dMRI but not visible in the connectivity atlas (white arrowheads).

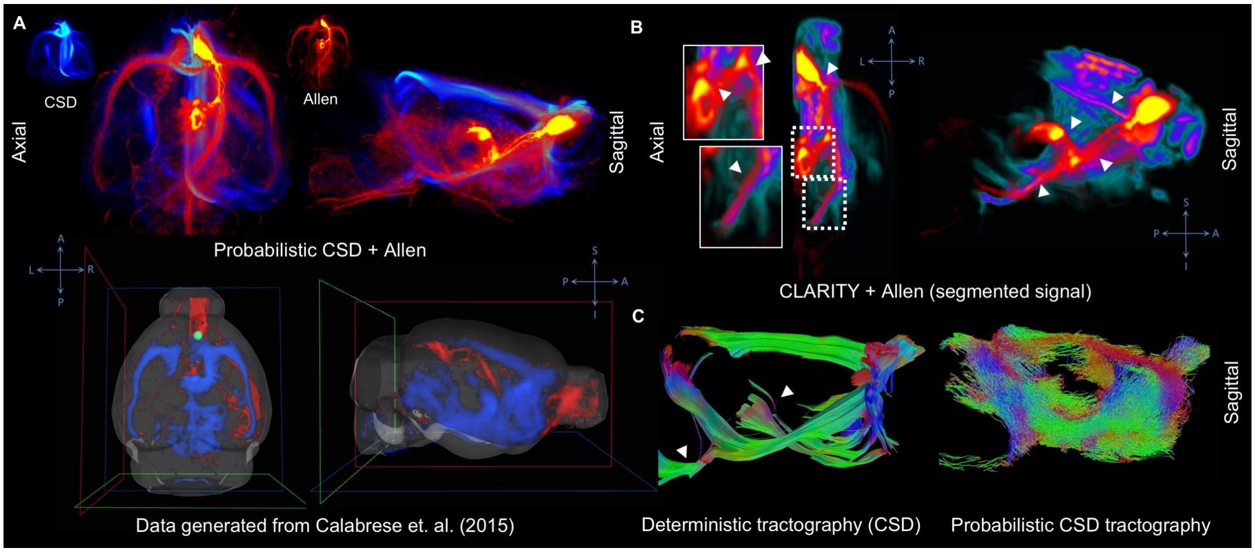

Supplementary Figure 11. Correspondence of efferent mPFC projections across different modalities.

(A) Comparison of our data and data generated from Calabrese et al (2015) (http://www.duhs.duke.edu/mouseconnectome) demonstrating the agreement between probabilistic dMRI (blue) and the Allen connectivity atlas (red) for mPFC projections, seeding (injecting in) from the PL area. It should be noted that the two dMRI datasets employ different probabilistic tracking algorithms. At the bottom blue (dMRI) is overlaid on red (Allen), while at the top red is overlaid on blue. A, Anterior; P, Posterior; L, Left; R, Right; S, Superior; I, Inferior.

(B) Projection density map of the Allen connectivity atlas experiment (red) overlaid on a registered projection density map of the segmented viral signal from the raw CLARITY volume (green). Both maps are thresholded to highlight areas of high signal density (virus uptake). The maps were smoothed to account for registration error. White arrowheads show areas of good agreement between the two modalities.

(C) Sagittal views of tractography streamlines contrasting two CSD-based tracking algorithms, deterministic (left) and probabilistic (right), of the same dMRI data and employing the same seed mask. White arrowheads show tracts not well depicted by deterministic tensor tractography.

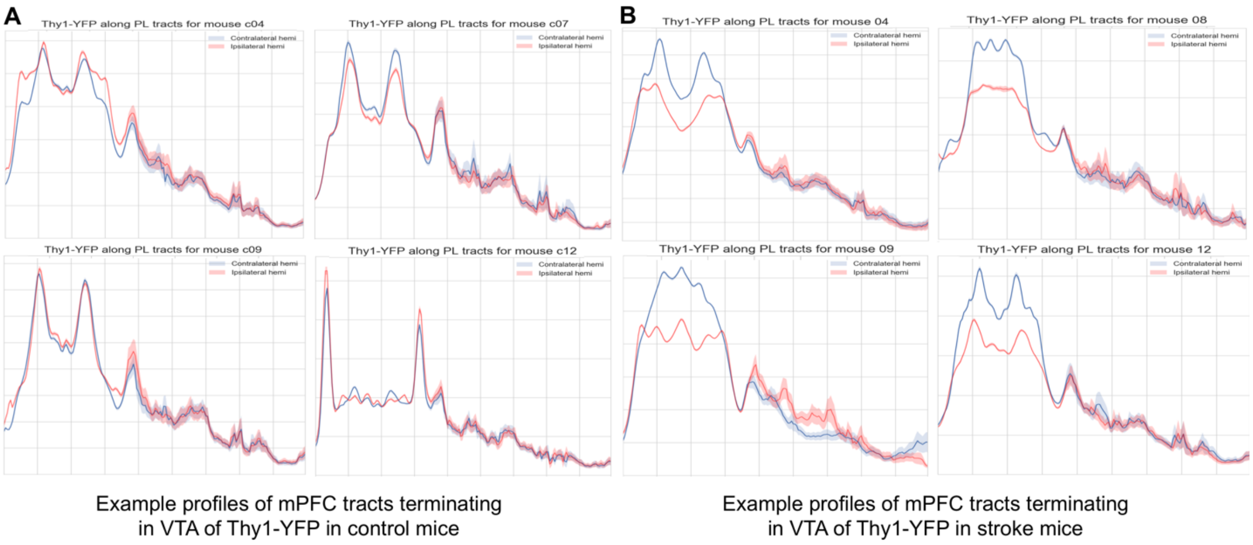

Supplementary Figure 12. Example profiles of Thy1-YFP in control and stroke mice along mPFC tracts using dMRI tractography.

(A) Tract profiles of Thy1-YFP intensity values along mPFC tracts terminating in VTA in four control mice in ipsilateral (red curves) and contralateral (blue curves) hemispheres. No substantial hemisphere differences are observed.

(B) Similar tract profiles in four stroke mice in the ipsilateral (red) and contralateral (blue) hemispheres to the stroke. Thy1-YFP intensities are decreased in the ipsilateral hemisphere compared to the contralateral hemisphere, highlighting cellular and fiber modulations due to stroke.

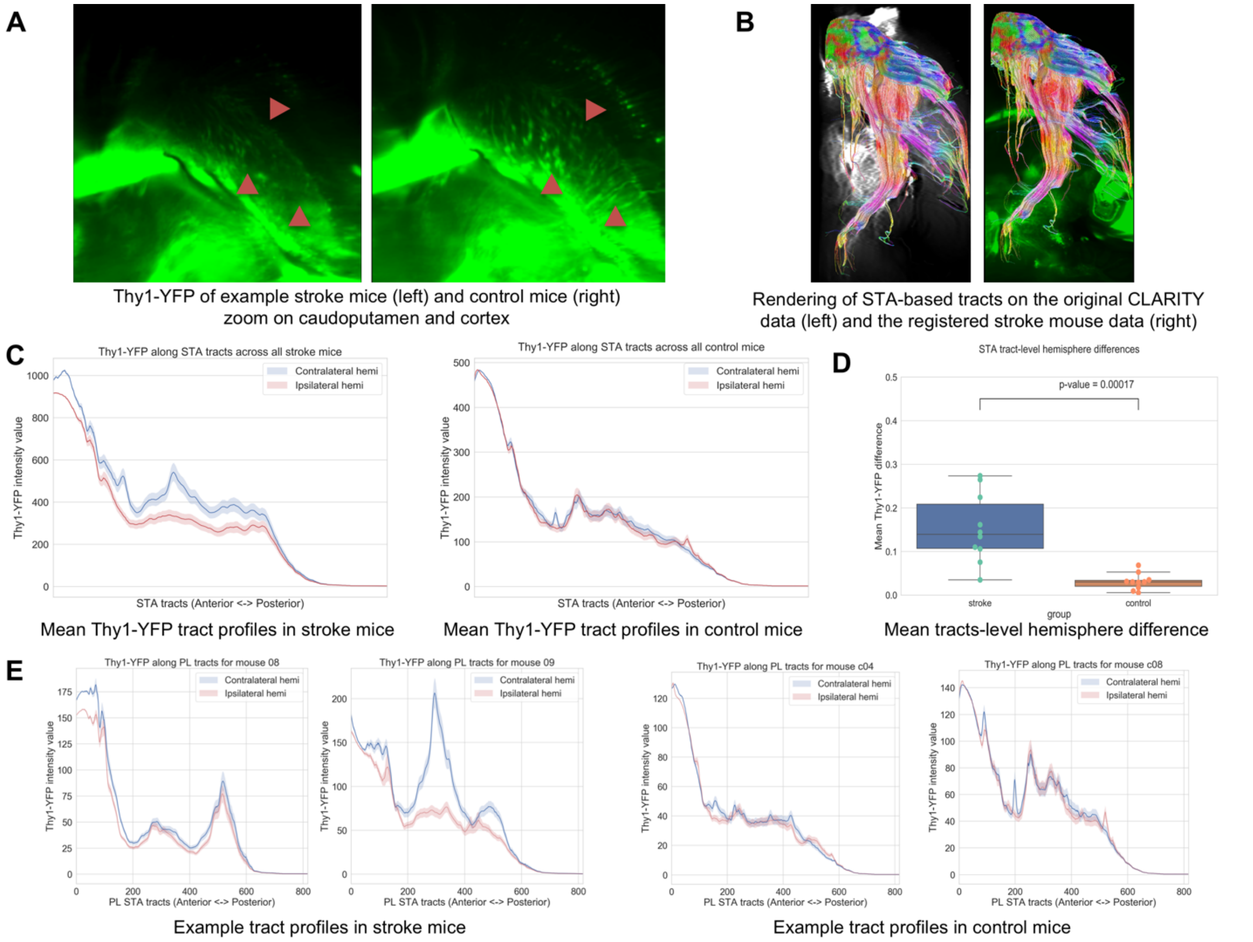

Supplementary Figure 13. Thy1-YFP cellular modulations along STA-based tractography in acute stroke.
(A) Thy1-YFP CLARITY axial images from a stroke mouse (left) showing lower staining and number of tracts in the caudoputamen and cortex compared to a control mouse (right).
B. Structure tensor analysis (STA)-based tractography overlaid on the original viral tracing CLARITY volume (left) and a registered stroke mouse volume (right).
(C) Mean tract-based Thy1-YFP intensity for the two tracts ipsilateral (red) and contralateral (blue) to the stroke, showing decrease ipsilateral Thy1-YFP along the tract in the stroke mice (left) but not the contralateral (right).
(D) Mean tract-level difference between contralateral and ipsilateral Thy1-YFP intensity in stroke (blue box) and control (orange box) mice. Center line of box plot represents the median, bounds represent the first and third quantiles, and whiskers represent the lowest and highest datum within 1.5x the interquartile range of the lower and upper quantiles.
(E) Example tract profiles for two stroke mice (left) and two controls (right).

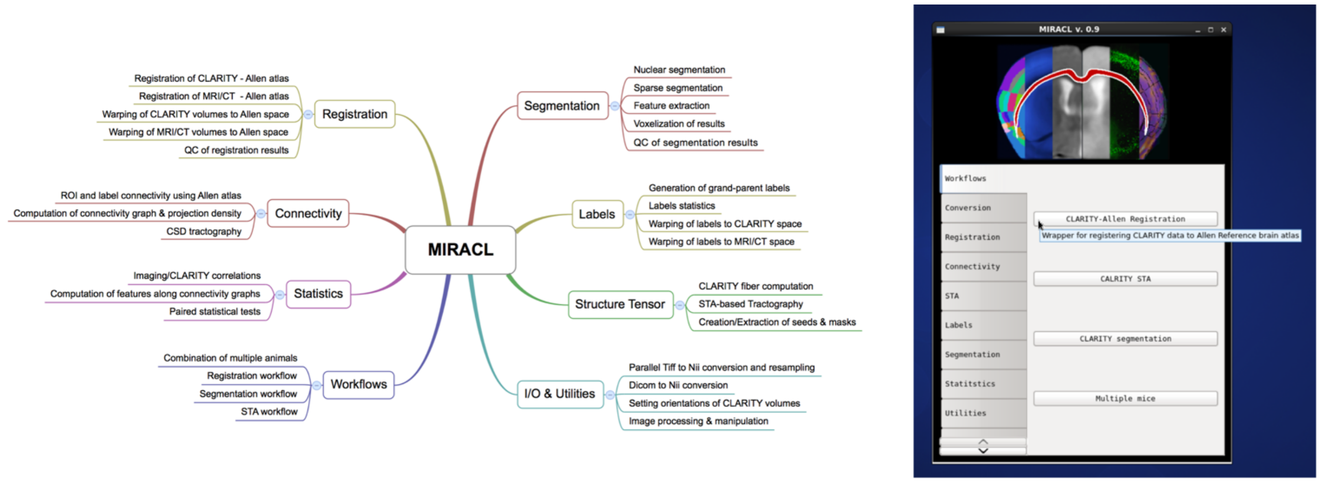

Supplementary Figure 14. Overview of the pipeline's core modules and functions and a snapshot of the graphical user interface.
