# Supplementary material for: Multimodal image registration and connectivity analysis for integration of connectomic data from microscopy to MRI
Source: Nat Commun. 2019 Dec 3;10:5504. doi: 10.1038/s41467-019-13374-0 (PMC6890789; doi:10.1038/s41467-019-13374-0)
Supplement: Supplementary file 2 — Description of Additional Supplementary Files [file 41467_2019_13374_MOESM2_ESM.docx]

**Description of Additional Supplementary Files**

**Supplementary Video 1.** Thy1-YFP and PI labeling in an example stroke mouse.

**Supplementary Video 2.** Registration of a green autofluorescence channel to the Allen regional atlas (ARA).

**Supplementary Video 3.** Whole-brain network graph of the entire Allen connectivity atlas.
