# Supplementary material for: Multimodal image registration and connectivity analysis for integration of connectomic data from microscopy to MRI
Source: Nat Commun. 2019 Dec 3;10:5504. doi: 10.1038/s41467-019-13374-0 (PMC6890789; doi:10.1038/s41467-019-13374-0)
Supplement: Supplementary file 6 — Reporting Summary [file 41467_2019_13374_MOESM6_ESM.pdf]

nature research

Corresponding author(s): Maged Goubran

Last updated by author(s): Jul 31, 2019

Reporting Summary

Nature Research wishes to improve the reproducibility of the work that we publish. This form provides structure for consistency and transparency in reporting. For further information on Nature Research policies, see Authors & Referees and the Editorial Policy Checklist.

Statistics

For all statistical analyses, confirm that the following items are present in the figure legend, table legend, main text, or Methods section.

n/a	Confirmed	
☐	☒	The exact sample size (*n*) for each experimental group/condition, given as a discrete number and unit of measurement
☐	☒	A statement on whether measurements were taken from distinct samples or whether the same sample was measured repeatedly
☐	☒	The statistical test(s) used AND whether they are one- or two-sided<br>*Only common tests should be described solely by name; describe more complex techniques in the Methods section.*
☐	☒	A description of all covariates tested
☐	☒	A description of any assumptions or corrections, such as tests of normality and adjustment for multiple comparisons
☐	☒	A full description of the statistical parameters including central tendency (e.g. means) or other basic estimates (e.g. regression coefficient) AND variation (e.g. standard deviation) or associated estimates of uncertainty (e.g. confidence intervals)
☐	☒	For null hypothesis testing, the test statistic (e.g. *F*, *t*, *r*) with confidence intervals, effect sizes, degrees of freedom and *P* value noted<br>*Give P values as exact values whenever suitable.*
☒	☐	For Bayesian analysis, information on the choice of priors and Markov chain Monte Carlo settings
☒	☐	For hierarchical and complex designs, identification of the appropriate level for tests and full reporting of outcomes
☐	☒	Estimates of effect sizes (e.g. Cohen's *d*, Pearson's *r*), indicating how they were calculated

Our web collection on statistics for biologists contains articles on many of the points above.

Software and code

Policy information about availability of computer code

Data collection	The raw CLARITY sections were imaged using an Olympus FV1200 system equipped with a 10x water-immersion objective (numerical aperture: 0.6; working distance: 3 mm; step size, 5 µm). They were acquired as 16-bit TIFF files. The images were further processed by blind 3D deconvolution using AutoQuantX3 (Media Cybernetics).<br><br>Histological slides were were captured using a CCD camera at 10x magnification (AxioCam MRm; Carl Zeiss AB, Switzerland), the Axio Imager M2 (Carl Zeiss AB, Switzerland), and the MBF software Neurolucida (MBF Bioscience, Williston, VT, USA).
Data analysis	We have made our pipeline open-access for the community. It is freely available at: https://github.com/mgoubran/MIRACLdev.<br><br>The presented pipeline is made up of custom code based on several open-source software, the Allen brain atlas resources and publicly available Python libraries.<br><br>The following open-source software were used:-<br>For registration: Advanced Normalization Tools (ANTs) [version 2.1], convert3D (c3d) [version 1.0].<br>For segmentation: Fiji (ImageJ) [version 2].<br>For connectivity analysis: Allen brain institute connectivity API<br>For diffusion MRI: Mrtrix3 [version 3], FSL [version 5.0.9].<br>ITKsnap [v 3.4] was used for stroke mask delineation and visualization.

For manuscripts utilizing custom algorithms or software that are central to the research but not yet described in published literature, software must be made available to editors/reviewers. We strongly encourage code deposition in a community repository (e.g. GitHub). See the Nature Research guidelines for submitting code & software for further information.

Data

Policy information about availability of data

All manuscripts must include a data availability statement. This statement should provide the following information, where applicable:

- Accession codes, unique identifiers, or web links for publicly available datasets
- A list of figures that have associated raw data
- A description of any restrictions on data availability

A subset of the datasets generated and analyzed during the current study will be released as examples with the pipeline, and more are available upon request.

Field-specific reporting

Please select the one below that is the best fit for your research. If you are not sure, read the appropriate sections before making your selection.

☒ Life sciences ☐ Behavioural & social sciences ☐ Ecological, evolutionary & environmental sciences

For a reference copy of the document with all sections, see nature.com/documents/nr-reporting-summary-flat.pdf

Life sciences study design

All studies must disclose on these points even when the disclosure is negative.

Sample size	For the middle cerebral artery occlusion (MCAO) model, we used n=10 Thy1-YFP mice, and n=10 wild-type littermates in the control group. All the mice underwent light-sheet imaging. All the stroke mice and three of the control mice underwent in-vivo MR imaging, ex-vivo CT, propidium iodide (PI) staining, and confocal microscopy.<br>For the medial pre-frontal cortex (mPFC) CLARITY viral tracing (CAPTURE labeling) we used n=3 wild-type (not part of the previous control cohort), which were injected with a 1ul AAV8-CaMKIIa-EYFP-NRN in area PL within the right mPFC (AP=2.0, L=0.3, V=2.5mm).<br><br>For the ultra-high-resolution ex-vivo MRI (diffusion and structural scans) we employed an excised control mouse brain (not part of the previous control cohorts), which was scanned for 2 days.<br><br>The sample sizes were deemed sufficient since this a technical study focused on presenting computational tools and the biological findings were used as a validation and proof-of-concept of the utility and impact of our pipeline.
Data exclusions	No data were excluded from the analyses
Replication	We performed a histological validation experiment on n=5 stroke mice.<br>We also tested our registration and segmentation algorithms on data not employed in the study, including data with intensity inhomogeneities and acquisition errors, and found similar fidelities to the presented results in the paper (examples shown in Suppl. Fig. 2 & 3).
Randomization	Not relevant to the study, as no treatment or conditional model was tested.
Blinding	The control mice were employed to compute normalized cell densities per brain region (Allen label). All the remaining analyses were performed on the stroke cohort. No blinding was necessary.

Reporting for specific materials, systems and methods

We require information from authors about some types of materials, experimental systems and methods used in many studies. Here, indicate whether each material, system or method listed is relevant to your study. If you are not sure if a list item applies to your research, read the appropriate section before selecting a response.

Materials & experimental systems

n/a	Involved in the study	
☐	☒	Antibodies
☒	☐	Eukaryotic cell lines
☒	☐	Palaeontology
☐	☒	Animals and other organisms
☒	☐	Human research participants
☒	☐	Clinical data

Methods

n/a	Involved in the study	
☒	☐	ChIP-seq
☒	☐	Flow cytometry
☐	☒	MRI-based neuroimaging

Antibodies

Antibodies used	Clarified tissues were incubated in Propidium Iodide (PI) solution (Cell Signaling Technologies) for 2-3 days, returned to PBST, incubated in RapiClear CS for 1 day and mounted using a Wilco dish.

Validation	For staining with two primary antibodies, histological sections were processed with the following steps: first washed in PBS, next incubated with pre-heated 0.1M sodium citrate at 60°C for 20 minutes for antigen retrieval. After that, sections were transferred to a blocking solution (10% normal animal serum, 1% bovine serum albumin in 0.3% PBS-triton X) for 1 hour, and incubated in a solution of primary antibodies for CD68 (1:500, Abcam ab53444) and microtubule associated protein 2 (MAP2) (1:200, D5G1, Cell Signaling Technology, Danvers, MA, USA) diluted in the blocking solution overnight at 4 °C. The next day, sections were washed in 0.3% PBS-triton X and incubated with secondary antibodies (1:500, Alexa fluor 546, Invitrogen A11081 for CD68; and 1:500, Alexa fluor 488, Invitrogen A32731 for MAP2, respectively) diluted in the blocking solution at room temperature for 2 hours. DAPI (1:2000) was added during the last 5 minutes of the secondary antibody incubation.

Animals and other organisms

Policy information about studies involving animals; ARRIVE guidelines recommended for reporting animal research

Laboratory animals	We used n=10 Thy1-YFP (line-H) mice, 8-10 weeks old, (B6.Cg-Tg(Thy1-YFP)15Jrs/JC57BL/6J, Jackson Laboratory) and n=10 wild-type littermates in the control group. All of the mice underwent light-sheet imaging of Thy1-YFP. Three of the 10 control mice underwent in-vivo MR imaging and PI staining. Mice were housed under a 12:12 hour light:dark cycle with food and water available ad libitum. For the histological validation experiment, we used N=5 mice.
Wild animals	n/a
Field-collected samples	n/a
Ethics oversight	All experiments were conducted in compliance with animal care laws and institutional guidelines, approved by the Stanford Institutional Animal Care and Use Committee, and in accordance with the guidelines from the NIH.

Note that full information on the approval of the study protocol must also be provided in the manuscript.

Magnetic resonance imaging

Experimental design

Design type	No functional MRI (fMRI) data were acquired.
Design specifications	*Specify the number of blocks, trials or experimental units per session and/or subject, and specify the length of each trial or block (if trials are blocked) and interval between trials.*
Behavioral performance measures	*State number and/or type of variables recorded (e.g. correct button press, response time) and what statistics were used to establish that the subjects were performing the task as expected (e.g. mean, range, and/or standard deviation across subjects).*

Acquisition

Imaging type(s)	Structural, relaxometry and diffusion MRI.
Field strength	7T
Sequence & imaging parameters	In-vivo: A T2-weighted FSE (fast spin echo) sequence with TE/TR=40/2500, a slice thickness = 0.5 mm and 0.1172x0.1172 mm in plane resolution was performed to get a high resolution structural image of the brain. A T2 map was acquired using a 2D SE (spin echo) sequence with TEs=12.1 ms, 24.3 ms, 36.4 ms, 48.5 ms, TR=3500 ms, slice thickness = 1mm, 0.5mm gaps between slices and 0.078x0.078 mm in plane resolution. Keeping the same geometry, a T1 map was acquired with a FSE-IR (fast spin echo with inversion recovery) sequence, TE/TR=7.2/5000 and TI=200, 400, 800, 1600, 3200 ms.<br><br>Ex-vivo: T1-weighted FLASH (fast low angle shot) sequence with TE/TR = 20/42.9, 8 averages and 100 µm isotropic resolution.
Area of acquisition	Whole-brain for both in-vivo and ex-vivo scans.

Diffusion MRI ☒ Used ☐ Not used

Parameters	In-vivo: The diffusion MRI scan consisted of an EPI (echo planar imaging) sequence with bipolar diffusion gradients, TE/TR = 16.3/1200, 4 averages, 0.1172x0.1172 mm in-plane resolution, 1mm slice thickness, 5mm gap between adjacent slices, 3 b=0 images and 18 linear independent diffusion directions with b=1000 s/mm2.<br><br>Ex-vivo: The diffusion scan had TE/TR = 28.8/500 ms, 200 µm isotropic resolution, 70 b=0 scans, 150 directions with b=1000 s/mm2, 230 directions with b=2000 s/mm2, 270 directions with b=4000 s/mm2 and 350 directions with b=8000 s/mm2.

Preprocessing

Preprocessing software	Processing scripts and routines developed in-house, based on Advanced Normalization Tools (ANTs) [version 2.1], convert3D (c3d) [version 1.0].

For diffusion MRI: Mrtrix3 [version 3], FSL [version 5.0.9].

ITKsnap [v 3.4] was used for stroke mask delineation and visualization.

preprocessing steps: intensity normalization, brain extraction, artifact removal.
group-heat maps: Gaussian smoothing.

Normalization

Non-linear (including an affine step) registration (normalization).

Normalization template

Allen mouse brain atlas.

Noise and artifact removal

Affine normalization between sequences to the high-resolution structural scan and eddy correction for diffusion MRI for motion artifacts.

Volume censoring

No volume censoring was performed.

Statistical modeling & inference

Model type and settings

Paired t-tests between regions (Allen labels) of both hemispheres.
Voxel-wise Spearman's rank correlations between MRI and CLARITY heat-maps, as well as between projection density maps of MRI, CLARITY and Allen connectivity atlas.

Effect(s) tested

Differences of means between labels of each hemispheres.
Multi-modal correlations of noninvasive imaging and CLARITY features.

Specify type of analysis: ☒ Whole brain ☐ ROI-based ☐ Both

Statistic type for inference
(See Eklund et al. 2016)

Voxel-wise (dMRI), no fMRI data acquired.

Correction

n/a

Models & analysis

n/a	Involved in the study
☐	☒ Functional and/or effective connectivity
☐	☒ Graph analysis
☒	☐ Multivariate modeling or predictive analysis

Functional and/or effective connectivity

No fMRI data were acquired. For connectivity analysis of CLARITY (not MRI) data we relied on measures of connectivity strength from viral tracing experiments (Allen connectivity atlas), specifically employing normalized projection density.

Graph analysis

Network graphs were computed for CLARITY (not MRI) data and no node summaries were used.
